# The effect of drought on physiological and agronomic traits of tef [*Eragrostis tef* (Zucc.) Trotter]

**DOI:** 10.3389/fpls.2026.1880325

**Published:** 2026-09-16

**Authors:** Beakal Tadesse Girma, Amsalu Gobena Roro, Kebebew Assefa Abebe, Zerihun Tadele, Jill Margaret Farrant

**Affiliations:** 1 Plant Stress Laboratory, Department of Molecular and Cell Biology, University of Cape Town, Cape Town, South Africa; 2 Ethiopian Institute of Agricultural Research, Addis Ababa, Ethiopia; 3 School of Plant and Horticultural Sciences, College of Agriculture, Hawassa University, Hawassa, Ethiopia; 4 Crop Breeding and Genomics, Institute of Plant Sciences, University of Bern, Bern, Switzerland; 5 Department of Plant Sciences, Bahir Dar University, Bahir Dar, Ethiopia

**Keywords:** agronomic traits, climate change, physiological traits, productivity, TEF, terminal drought, water use efficiency

## Abstract

Increased intensity and duration of drought as a consequence of global warming is resulting in increased crop failure and food insecurity. This is particularly prevalent in regions where agriculture is rain fed, as the resultant water deficit in crop tissues as a consequence of insufficient soil moisture to sustain metabolic integrity results in subcellular damage and, ultimately tissue death. Different plants can perceive moisture stress stimuli from their environment and develop adaptive mechanisms to cope with water stress. However, the response of tef [*Eragrostis tef* (Zucc.) Trotter], a staple food crop for millions of people in Ethiopia, to varying soil moisture conditions has not been adequately investigated. Therefore, the current study was designed to identify drought-tolerant tef genotypes and their physiological and agronomic adaptation mechanisms of tolerance to moisture deficit at the terminal phenological stage of the plant which aligns to the flowering period. A total of 20 tef genotypes were evaluated for their response to physiological and agronomic traits under well-watered and water-deficit conditions. The results showed significant variation among genotypes, moisture regimes, and their interactions for most measured traits. However, canopy temperature and shoot biomass did not exhibit a significant genotype × moisture regime interaction. In addition, chlorophyll content, normalized difference vegetation index, grain yield, and yield components were significantly reduced by moisture deficit. Although canopy temperature increased under both moisture conditions, the average increase was more pronounced under water-deficit (6.7 °C) than under well-watered (4.7 °C) conditions. Tef genotypes such as Boni, Dtt-2, and Enatite showed higher water use efficiency and intermediate stomatal conductance, indicating adaptive strategies that minimize water loss while maintaining efficient utilization of available soil moisture for metabolic processes, thereby contributing to higher grain yield under stress conditions. The study indicated that tef genotypes employ diverse physiological and agronomic mechanisms to overcome the adverse effects of drought during the flowering or reproductive stage of the plant. However, further studies evaluating diverse genotypes across multiple environments and seasons are needed to validate the findings of the current study. Integrating additional physiological traits, particularly root characteristics, is needed for the development of reliable traits for breeding.

## 1 Introduction

Climate change is significantly and negatively affecting agricultural production where drought is one of the major consequences thereof (Dai, 2013; El-Hendawy et al., 2017; Sallam et al., 2019). The reduction is not only due to climatic factors but also due to the interaction between a variety of abiotic and biotic stresses (Salehi-Lisar and Bakhshayeshan-Agdam, 2016; Hussain et al., 2018). However, morphological and physiological modifications against drought were observed in drought tolerant species that enable them to cope with water-limited conditions. One potential strategy for mitigating drought stress is the utilization of orphan crops, which are naturally adapted to marginal environments and capable of sustaining normal physiological growth and productivity under drought conditions (Farrant and Hilhorst, 2022). Among these crops, tef [*Eragrostis tef* (Zucc.) Trotter], an indigenous crop from Ethiopia, is considered as the crop of choice due to its relatively higher tolerance to water deficit (Tadele, 2019). Tef exhibited moderate drought tolerance compared with two related wild species: the tolerant *E. curvula* and the susceptible *E. capensis* (Balsamo et al., 2006). The crop has a potential to withstand up to 50-60% loss of total water content (Kamies et al., 2017).

Despite its general adaptability, tef remains sensitive to moisture deficit stress, with productivity markedly reduced when soil moisture declines by more than 25% (Ali, 2013; Yihun, 2015). Although the sensitivity of tef to drought varies based on the growth cycle of the plant, moisture stress during the flowering period results in pronounced negative effect on grain yield (Takele, 1997; Admas and Belay, 2011). Such yield reduction in tef due to moisture scarcity might be associated with the reduction in flower fertility, delay in initiation of panicle and grain filling (Mengistu, 2009). Drought at post flowering stage commonly referred to as “terminal drought” and stress at this stage shown to be a major determinant of grain yield in tef (Abraha, 2016).

Previous studies on sunflower indicated that the growth stage of the plant at which drought is imposed could influence plant architecture and ultimately resource utilization of a given genotype (Casadebaig et al., 2008). On other hand, studies on tea plant and maize showed that thickening of cuticles, reduced gas exchange through down regulation of stomatal dynamics and reduction of non-stomatal transpiration are key physiological parameters significantly responding to moisture stress and serve as indicators of drought resistance (Ristic and Jenks, 2002; Chen et al., 2020). Crops like sorghum with high adaptation plasticity to drought are known to alter their osmotic balance and stomatal conductance following the fluctuation of soil moisture (Abreha et al., 2022).

Despite efforts to investigate the effects of drought on the morphological traits of tef (Shiferaw et al., 2012b; Abraha, 2016; Ferede et al., 2018), studies with an emphasis on physiological mechanisms underlying drought tolerance remain limited. Existing physiological studies have not comprehensively integrated gas exchange characteristics with photosynthetic components and morphological traits and have largely been conducted under controlled conditions (Mengistu, 2009; Alemu et al., 2024), limiting their practical relevance. Field-based investigations are scarce, and most existing studies rely only on natural rainfall conditions (Abraha, 2016). Such conditions are highly variable and limit the exact understanding of the nature of drought stress at various stages in the life cycle. Furthermore, the lack of controlled water management constrains the regulation of drought severity. To maneuver this the current study targeted the mid of rainy season based on the metrology data to both the use of advantage of the rainfall at earlier stage and synchronize the drought stressed plots with the drought while it is supplemented by irrigation.

A further constraint to our understanding of tef’s drought tolerance is that few studies have adequately incorporated improved tef varieties with diverse growth habits and maturity periods. Consequently, the physiological mechanisms underlying drought adaptation in improved genotypes remain poorly understood, particularly under the increasing pressures of climate change. In addition, comprehensive time-series studies that integrate physiological and agronomic traits in newly developed improved varieties are largely lacking. The genotypes used in the current study include recently improved drought-tolerant lines (Cannarozzi et al., 2018; Kebede et al., 2022). Combining physiological indicators facilitates the screening and selection of diverse genotypes in breeding programs aimed at improving drought tolerance. Furthermore, this approach provides a more comprehensive understanding of physiological and agronomic drought adaptation mechanisms under field conditions by elucidating the relationships between moisture scarcity, gas exchange, canopy temperature, photosynthetic components, and associated agronomic responses.

Therefore, the present study was designed to investigate the agronomic and physiological mechanisms underlying drought adaption in selected tef genotypes, including newly developed lines under field conditions. Key physiological and agronomic traits associated with biomass production and grain yield were evaluated under contrasting moisture conditions. Thus, this study aims to both identify drought tolerant tef genotypes and determine how these genotypes adapt to terminal drought stress.

## 2 Materials and methods

The study was conducted at Hawassa University farm, Hawassa, Ethiopia, from October 2023 to January 2024. Hawassa is located 273 km south of Addis Ababa at 7° 03’ N, 38° 30’ E and at an altitude of 1650 meters above sea level. Prior to the experiment, soil samples were collected from 18 random points at a depth of 0–30 cm and combined into six composite samples. The chemical and physical properties of the soil were determined according to the protocol described by Tegegn (2019). The experimental site has well-drained loam soil (**Supplementary Table S1**). Meteorological data such as daily rainfall and temperature during the experimental period for the study area were obtained from National Meteorology Agency (NMA) of Ethiopia. While the average maximum temperature during the growing season was 28.2°C, the average minimum temperature was 14.1 °C and the total rainfall during the cropping season was 132.6 mm (**Supplementary Figure S1**).

For this study, twenty tef genotypes were used (**Table 1**). These genotypes were obtained from the National Tef Improvement Program of the Ethiopian Institute of Agricultural Research and the Tef Improvement Project of the University of Bern. The genotypes were selected from the previous set of 60 diverse genotypes following their evaluation under drought stress and optimum moisture conditions at two locations during the terminal growth stage.

**Table 1:** Twenty genotypes evaluated in the experiment and seed source.

SN	Genotype	SN	Genotype
1	DZ-01-1825^†^	11	Ajora (PGRC/E 205396) ^†^
2	Genete (DZ-01-146) ^†^	12	Tsedey (DZ-Cr-37) ^†^
3	Boni (DZ-Cr-498 RIL-37) ^†‡^	13	Dega Tef (DZ-01-2675) ^†^
4	DZ-01-179 ^†^	14	Gimbichu (DZ-01-899) ^†^
5	Dukem (DZ-01-974) ^†^	15	Enatite (DZ-01-354) ^†^
6	Yilmana (DZ-01-1868) ^†^	16	RIL-44 ^†‡^
7	Koye (DZ-01-1285) ^†^	17	RIL-94 ^†^
8	Boset (DZ-Cr-409 Sel 50D) ^†^	18	DZ-01-951 ^†^
9	RIL-302 ^†‡^	19	Dtt-2 ^‡^
10	Alba ^†^	20	TDT-4-15 ^‡^

Where: genotypes designated by † are sourced from EIAR: Ethiopian Institute of Agricultural Research, genotypes designated by ‡are sourced from UB, University of Bern, and genotypes designated by †‡ are mutant lines developed by UB and cross made with adaptable lines by EIAR.

### 2.1 Experimental design and water regimes

The experiment was arranged in a 5×4 alpha lattice design with three replications (see the field layout **Supplementary Figure S2**). Each incomplete block consisted of five plots with the area of 0.5m². Each plot consisted of five rows/lines, each 0.5 m long and spaced 0.2 m apart, with 0.5 m spacing between plots. A small plot size was adopted to allow the inclusion of a larger number of genotypes, increase the number of replications, and facilitate the evaluation of contrasting moisture conditions while reducing spatial variability among plots. However, because of the small plot size, the entire plot was harvested rather than excluding the border rows. This approach was considered appropriate because each plot had its own border rows, and a uniform distance was maintained between adjacent plots. Furthermore, no extreme environmental events, such as flooding, or biotic stress, occurred during the experiment that could have conferred an advantage or disadvantage to the border rows. Therefore, harvesting the entire plot was unlikely to introduce systematic bias into the estimation of yield or other traits measured on a whole-plot basis. In each plot, 0.5 g of seed was drilled across the five rows/lines. Fertilizer was applied according to regional recommendations: 22 kg ha^-1^ of urea (46% N) being applied half at the sowing and the remaining half at the beginning of tillering, and 158 kg ha^-1^ NPS (19% N, 38% P2O5 and 7% S) being applied at the sowing (Fikere et al., 2020). The irrigation frequency and amount were determined using CROPWAT 2.0 (Yihun, 2015). During the cropping season, a total of 155 mm of water was applied using supplemental irrigation and 132.6 mm was received from the rainfall. No rainfall occurred during the heading and later stages.

To induce moisture stress, irrigation was withheld from half of the plants of each variety at the heading stage (approximately 42–51 days after sowing) until harvest. The other half remained irrigated (control), and all plants were harvested at physiological maturity. These will be termed as water-deficit (WD), or well-watered (WW) conditions respectively. However, it should be noted that the two moisture conditions were spatially separated by 2 m to avoid potential spillover effects during irrigation.

### 2.2 Data collection

#### 2.2.1 Soil and leaf water content

For both WW and WD conditions, soil volumetric water content (VWC) and leaf relative water content (RWC) were determined gravimetrically at two time points: at the onset of drought stress (heading stage) and two weeks after irrigation cessation.

Soil VWC was determined at a depth of 0–30 cm below ground level, using the following formula:


$$VWC(\%)=\frac{fresh\;mass-dry\;mass}{dry\;mass}*100$$

Soil samples were collected by taking three subsamples from each replication, one from each edge and one from the center of each field plot. The three subsamples were then composited into a representative soil sample for each replication under each moisture condition. Consequently, three composite soil samples were obtained for each moisture condition, representing the soil moisture status under WW and WD conditions.

Leaf RWC was determined as described by Vander Willigen et al. (2001) in which the fresh mass of individual leaves (n=1 per plot/n=3 per genotype and condition) was determined on harvest and after submerging in distilled water for 48 hours in the dark to obtain turgid mass. Samples were subsequently oven-dried at 70 °C for 72 hours to obtain dry mass. The RWC was calculated using the formula:


$$RWC(\%)=\frac{fresh\;mass-dry\;mass}{turgid\;mass-\;dry\;masss}*100$$

#### 2.2.2 Physiological parameters

Phenological, agronomic, and physiological data were collected both at the plot and individual plant level. Physiological data were collected weekly from the heading stage for four consecutive weeks starting from the onset of drought, on an individual plant basis. Chlorophyll content (CC) was measured using handheld portable SPAD (SPAD-502, Minolta Corp.) from three plants. The measurement using SPAD provides an indirect estimation of CC by determining the chlorophyll index, which is a reliable and non-destructive method for repeated measurements of leaf tissues over time (Vijayalakshmi et al., 2024). Normalized Difference Vegetation Index (NDVI) was measured using a handheld Green Seeker (Trimble Navigation Limited, USA), and canopy temperature (CT) was measured using a handheld infrared thermometer (IR-GUN-S, ThermoWorks Inc., USA). Measurements were taken from the three central rows of each plot and averaged at the same growth stage. Gas exchange parameters such as stomatal conductance (gs), assimilation rate (A), transpiration rate (E) and water use efficiency (WUE), were measured using a portable CO2/H2O analyser (CIRAS-3, PP Systems, Inc., Amesbury, USA) at end of the fourth week after cessation of irrigation (For details **Supplementary Table S2**).

#### 2.2.3 Phenological and agronomic traits

Phenological data, such as days to heading and maturity were recorded on a plot basis. Days to heading (DH) was defined as the number of days from sowing to 50% of the panicle emerging from the flag leaf. Days to maturity (DM) is the number of days from sowing to physiological maturity. Tef’s maturity is indicated by the yellowing of the stalks bearing the spikelets. These data were taken when 80% of the plants in a single experimental unit (plot) reached the desired stage. At crop maturity, agronomic data such as shoot biomass (SB), root biomass (RB), grain yield (GY), and thousand kernel weight (TKW) were collected according to the description provided in Chanyalew et al. (2021) (**Supplementary Table S3**).

### 2.3 Data analysis

The homogeneity of variance was tested prior to the analysis of variance (ANOVA). The data were then subjected to ANOVA for the 5×4 alpha lattice design using R version 4.3.3 (R Core Team, 2024). Both individual and combined ANOVA were applied to determine the effect of genotypes, moisture conditions and their interaction. Mean comparisons were carried out using the Least Significance Difference (LSD) test to determine the critical difference between tef genotypes, the moisture conditions and genotypes across moisture regimes. In addition, the percentage reduction for each trait was calculated for each genotype by comparing values under WD and WW conditions to quantify the extent of reduction in response to moisture scarcity relative to the WW conditions. In cases where the assumption of homogeneity of variance was violated, nonparametric Kruskal-Wallis tests were conducted (Kruskal and Wallis, 1952; Conover and Iman, 1979). Correlation analysis was conducted to examine the relationships between traits. Principal component analysis (PCA) was then conducted to assess the relationships among traits, examine the distribution and clustering of genotypes, and identify the major sources of variation under WD conditions using agronomic and physiological data collected. The standard deviation of physiological traits, including NDVI, CC (SPAD value), and CT, was calculated using weekly data collected under WD conditions from the onset of drought to four weeks after drought imposition. This analysis was performed to assess the relative stability of these traits among genotypes in response to increasing drought severity. Graph Pad Prism 10.5.0 (GraphPad Software, Boston, Massachusetts, USA) was used to generate the figures.

## 3 Results

### 3.1 Effects on soil and water content

Twenty tef genotypes were evaluated under two moisture regimes: WW and WD. Plants under WW conditions maintained a constant soil VWC of 37% and this was significantly reduced (P<0.05) to 15.7% after two weeks of withholding water (**Figure 1A**).

**Figure 1:**
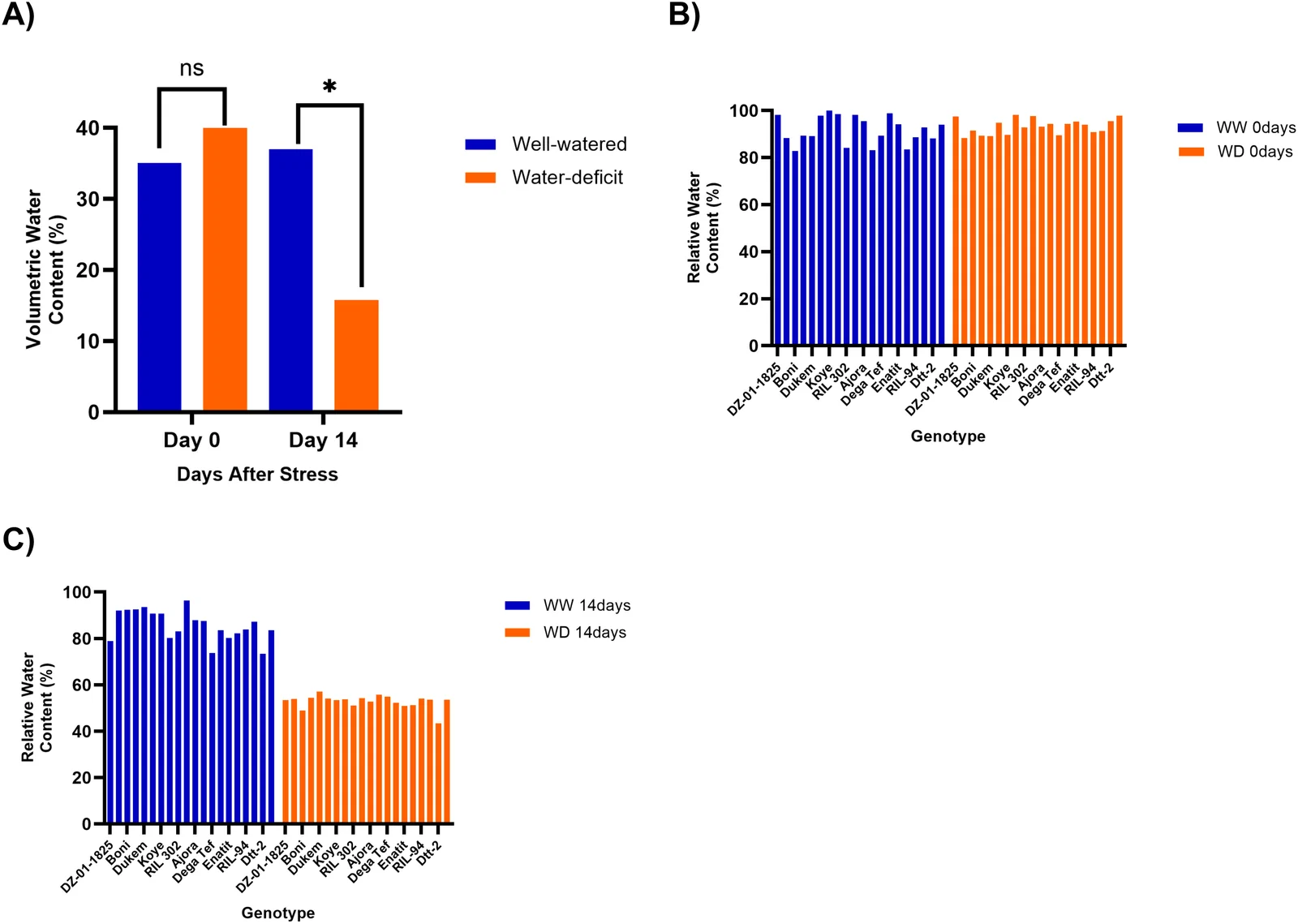
Change in soil and leaf water content at Day 0 (beginning of stress) and Day14 after moisture stress between well-watered (WW) and water-deficit (WD) conditions. **(A)** soil volumetric water content (VWC); **(B)** leaf relative water content (RWC) at Day 0; and **(C)** RWC at Day 14. Mean comparisons in panels **(B)** and **(C)** were conducted between WW and WD conditions; no significant difference was observed on Day 0 [panel **(B)**], whereas a significant difference was observed at Day 14 [panel **(C)**] between WW and WD conditions. P < 0.05; where: ns, non-significant.

Although leaf RWC values were similar for all 20 tef genotypes on Day 0 (**Figure 1B**), plants subjected to the WD conditions showed a significant reduction (P < 0.05) in RWC after 14 days of moisture stress, from 85.6% to 52.8% (**Figure 1C**). This substantial reduction in the RWC of tef plants exposed to two weeks of moisture scarcity indicates that almost all 20 tef genotypes investigated in the current study reduced leaf water content in times of moisture scarcity.

### 3.2 Effects on physiological traits

On average, NDVI values measured four weeks after dehydration showed a significant reduction (P < 0.05) in response to changes in moisture regime, from 0.41 in WW to 0.32 in WD conditions (**Table 2**; **Supplementary Table S4**). This indicates that considerable stress occurs during moisture deficit. In addition, significant differences in NDVI were observed among tef genotypes under both moisture conditions, suggesting differential responses among them (P < 0.001; **Supplementary Table S5**).

**Table 2:** Mean comparison for physiological traits of 20 tef genotypes between well-watered (WW) and water-deficit (WD) conditions.

Genotype	NDVI			CC (SPAD value)			CT (°C)		
	WW	WD	% R	WW	WD	% R	WW	WD	% I
DZ-01-1825	0.37^a^	0.31^b^	19.3	40.58^a^	30.60^b^	24.5	30.97^b^	32.69^a^	5.5
Genete	0.30^a^	0.23^b^	30.4	37.57^a^	27.57^b^	26.6	31.33^b^	34.26^a^	9.3
Boni	0.71^a^	0.38^b^	86.8	39.56^a^	32.22^b^	18.5	29.73^b^	31.39^a^	5.4
DZ-01-179	0.36^a^	0.30^b^	20.0	40.47^a^	32.60^b^	19.4	32.00^a^	32.90^a^	2.8
Dukem	0.34^a^	0.27^b^	25.9	36.81^a^	35.03^a^	4.8	30.87^b^	32.21^a^	4.3
Yilmana	0.37^a^	0.30^b^	23.3	38.49^a^	24.10^b^	37.4	31.07^b^	33.70^a^	8.5
Koye	0.36^a^	0.30^b^	20.0	45.44^a^	31.70^b^	30.2	31.07^a^	32.32^a^	4.0
Boset	0.33^a^	0.27^b^	22.2	27.68^a^	26.28^a^	5.0	30.90^b^	33.67^a^	8.9
RIL-302	0.46^a^	0.38^b^	21.0	43.14^a^	34.76^b^	19.4	30.37^b^	32.57^a^	7.2
Alba	0.43^a^	0.33^b^	30.3	36.40^a^	26.39^b^	27.5	32.63^b^	36.36^a^	11.4
Ajora	0.43^a^	0.31^b^	38.7	36.90^a^	30.51^b^	17.3	31.60^b^	35.23^a^	11.5
Tsedey	0.51^a^	0.44^b^	15.9	40.64^a^	33.46^b^	17.6	29.93^b^	32.71^a^	9.3
Dega Tef	0.38^a^	0.34^a^	11.7	42.37^a^	35.13^b^	17.0	32.47^b^	34.20^a^	5.0
Gimbichu	0.38^a^	0.29^b^	31.0	36.28^a^	27.69^b^	23.6	32.83^b^	35.63^a^	8.5
Enatite	0.45^a^	0.39^b^	15.4	39.18^a^	36.26^a^	7.4	28.60^b^	30.09^a^	5.2
RIL-44	0.51^a^	0.43^b^	18.6	37.03^a^	20.40^b^	44.9	28.83^b^	32.51^a^	12.7
RIL-94	0.44^a^	0.37^b^	18.9	38.64^a^	33.80^a^	12.5	30.33^b^	32.59^a^	7.4
DZ-01-951	0.35^a^	0.28^b^	25.0	35.00^a^	26.19^b^	25.2	30.33^b^	32.46^a^	7.0
Dtt-2	0.39^a^	0.29^b^	34.4	38.13^a^	33.94^a^	10.9	30.07^b^	32.04^a^	6.5
TDT-4-15	0.36^a^	0.29^b^	24.1	40.36^a^	28.41^b^	29.6	30.40^b^	32.50^a^	6.9
Mean	0.41	0.32	26.6	38.53	30.36	20.9	30.82	33.10	7.4
Max	0.71	0.44		45.44	36.26		32.83	36.36	
Min	0.30	0.23		27.68	20.40		28.60	30.09	
LSD (5%)	0.056			4.94			1.29		

Where: NDVI, normalized difference vegetation index; CC, chlorophyll content (SPAD value); CT, canopy temperature; %R, precent reduction; % I, percent increase; Max, maximum; Min, minimum; and LSD, least significant difference. Mean comparisons were conducted for individual traits under the two moisture conditions using the LSD test. Genotypes designated by the same letter are not significantly different at P<0.05 in their response to changes in moisture conditions. Although the LSD comparison was performed across all genotypes within both moisture conditions, labeling was specifically applied to facilitate comparison of genotype performance between the two moisture regimes. Therefore, the reported LSD value applies to all genotypes and moisture conditions for each respective trait. Mean comparisons among genotypes within each individual moisture conditions are provided in the **Supplementary Materials** (**Supplementary Table S5**).

Tef genotypes with high NDVI values under WD conditions include Tsedey, RIL-44, RIL-302, and Boni. In contrast, under WW conditions, higher NDVI values were observed for Boni, Tsedey, and RIL-44 (**Table 2**; **Supplementary Table S4**). Genotypes such as Dega Tef, Enatite, RIL-44, and RIL-94 showed relatively lower percentage reductions under WD conditions compared with WW conditions. The consistently high NDVI value of RIL-44, together with its smaller percentage reduction in WD conditions compared with WW conditions, indicates that this genotype is among the best in both moisture regimes.

Hand-held spectral absorbance device (SPAD) was used to determine the total CC in the leaves of the 20 tef genotypes under the two moisture regimes. The CC of leaves estimated using SPAD showed a significant reduction (P<0.05) in plants under WD conditions compared to those under WW conditions (**Supplementary Table S4**). In addition, significant differences (P<0.05) in CC were observed among genotypes within each moisture conditions, as well as their interaction (**Supplementary Tables S4**, **S6**). Significant interaction of genotypes and moisture conditions indicates inconsistent performance of the tef genotypes across the two moisture levels meaning that the genotype performing well in WW conditions may not do the same in WD conditions. This could be the difference in adaptation of genotypes to different environmental conditions which shows there need to be different selection and breeding strategies considering the environments separately (Mohammadi and Amri, 2021). However, the difference in CC between WW and WD conditions was not significant (P<0.05) for a few genotypes, including Dukem, Boset, and Enatite (**Table 2**). These genotypes also showed relatively lower percentage reduction in WD conditions compared with WW conditions, indicating their ability to maintain their chlorophyll under prolonged drought stress.

Tef genotypes with high CC values under WD conditions were Enatite, Dukem, and RIL-302. In contrast, under WW conditions, higher CC values were recorded for Koye and RIL-302 (**Supplementary Table S4**). This shows that RIL-302 maintained the highest CC under both WW and WD conditions although it was lowered by 27.7% under WD compared to WW conditions.

With exception of DZ-01–179 and Koye, CT was significantly higher (P < 0.05) for plants under WD conditions compared to those under WW conditions (**Table 2**). The lowest CT under WD conditions was recorded for Enatite (30.1 °C), Boni (31.4), Dtt-2 (32.0), and Koye (32.3). In contrast, under WW conditions, CT values were 28.6 °C for Enatite, 28.8 for RIL-44, and 29.7 for Boni. Although higher CT was recorded under WD compared to WW conditions, the differences among most genotypes were small, indicating that most genotypes maintain their CT when exposed to drought conditions. Hence, physiological processes such as photosynthesis are likely not substantially altered. However, genotypes such as DZ-01-179, Koye, Enatite, Dega Tef, and Boni showed lower percentage increases in CT under WD conditions compared with WW conditions. Among these, Enatite and Boni combined lower CT under WD conditions with smaller percentage changes relative to WW conditions, indicating their capacity to better maintain CT under prolonged drought stress.

Change in physiological parameters at different phases of drought. In addition to measurements taken four weeks after the onset of drought, physiological parameters, such as NDVI, CC, and CT, were also measured at the onset of drought and on weekly basis for three weeks following the last irrigation under WD conditions (Day 28), while genotypes under WW conditions continued to receive regular irrigation.

Overall, NDVI and CC decreased as drought progressed (**Figures 2**, **3**). In contrast, CT increased during this period under WD conditions (**Figure 4**). Under WW conditions, NDVI and CC initially increased and then declined by the end of the four-week period, whereas CT showed a consistent increase throughout.

**Figure 2:**
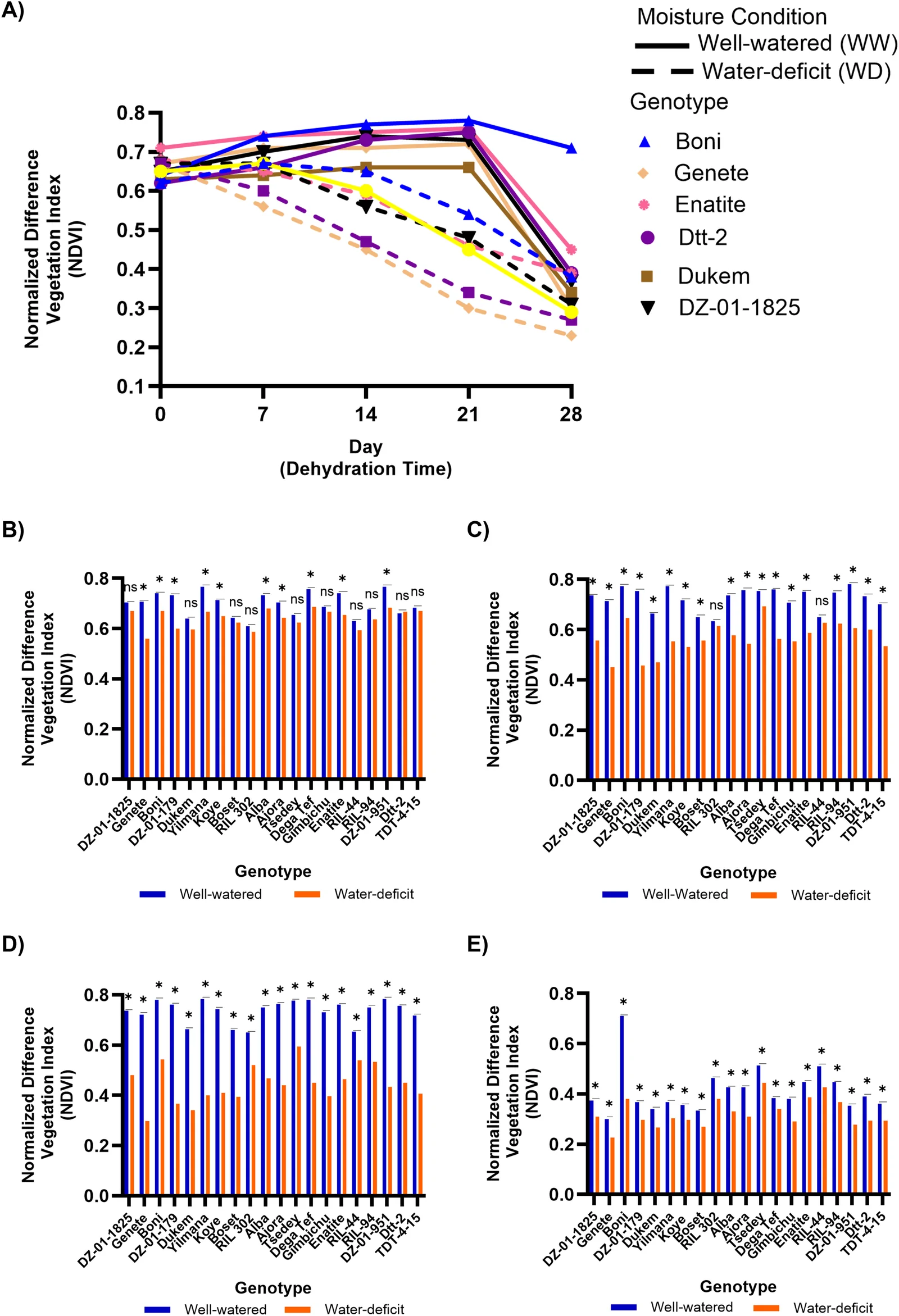
Normalized difference vegetation index (NDVI) for tef genotypes under well-watered (WW) and water-deficit (WD) conditions. **(A)** for six selected genotypes across four weeks; **(B)** on Day 7; **(C)** Day14; **(D)** Day 21; and **(E)** Day 28 after withholding water. Mean comparisons in panels **(B–E)** were conducted for individual genotypes between WW and WD conditions; where: *significant difference at P<0.05 and ns, non-significant.

**Figure 3:**
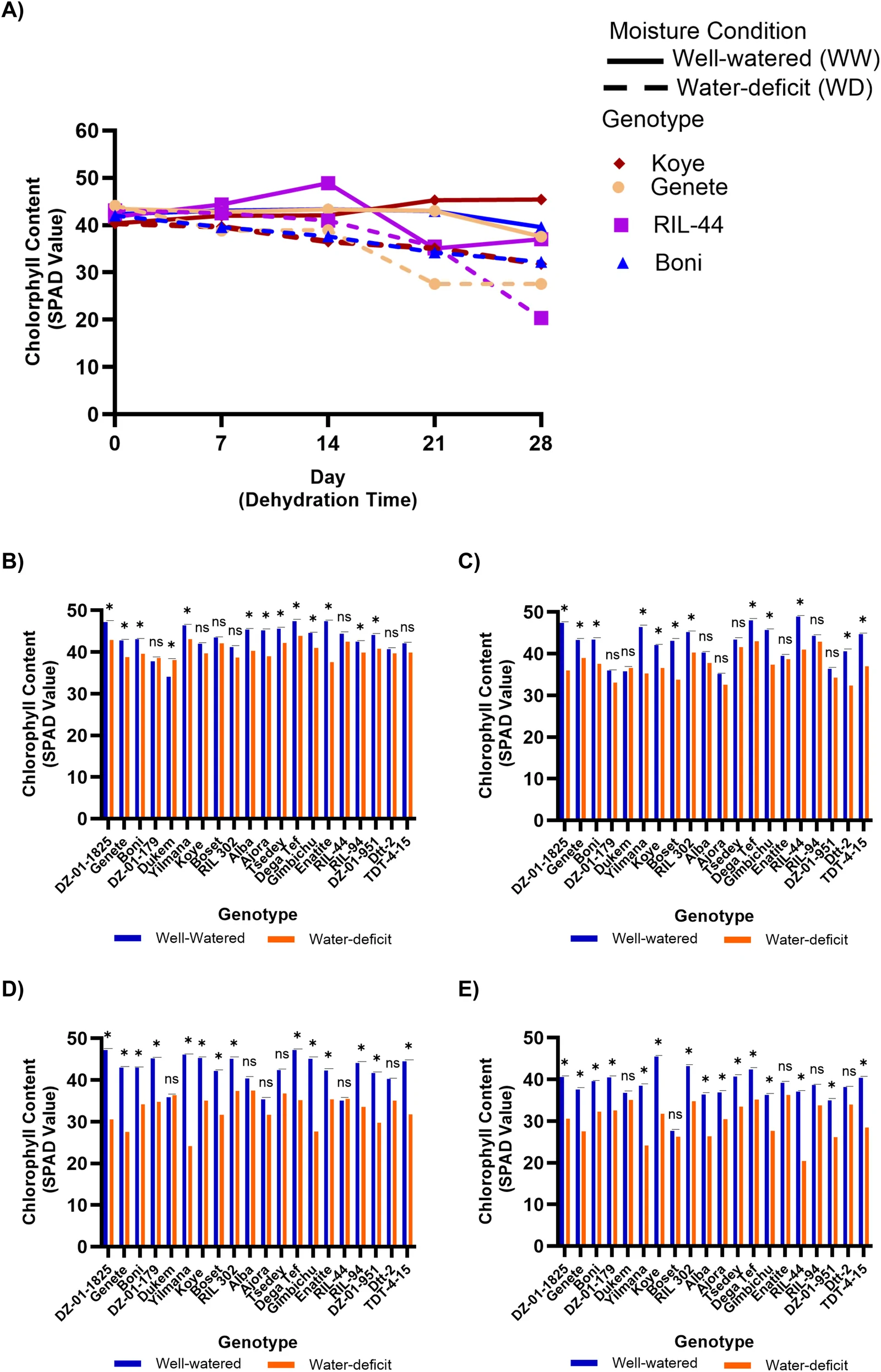
Chlorophyll content (CC) for tef genotypes under well-watered (WW) and water-deficit (WD) conditions. **(A)** across four weeks for four selected genotypes; **(B)** on Day 7; **(C)** Day 14; **(D)** Day 21; and **(E)** Day 28 after beginning of drought stress. Mean comparisons in panels **(B–E)** were conducted for individual genotypes between WW and WD conditions; where: *significant difference at P<0.05 and ns, non-significant.

**Figure 4:**
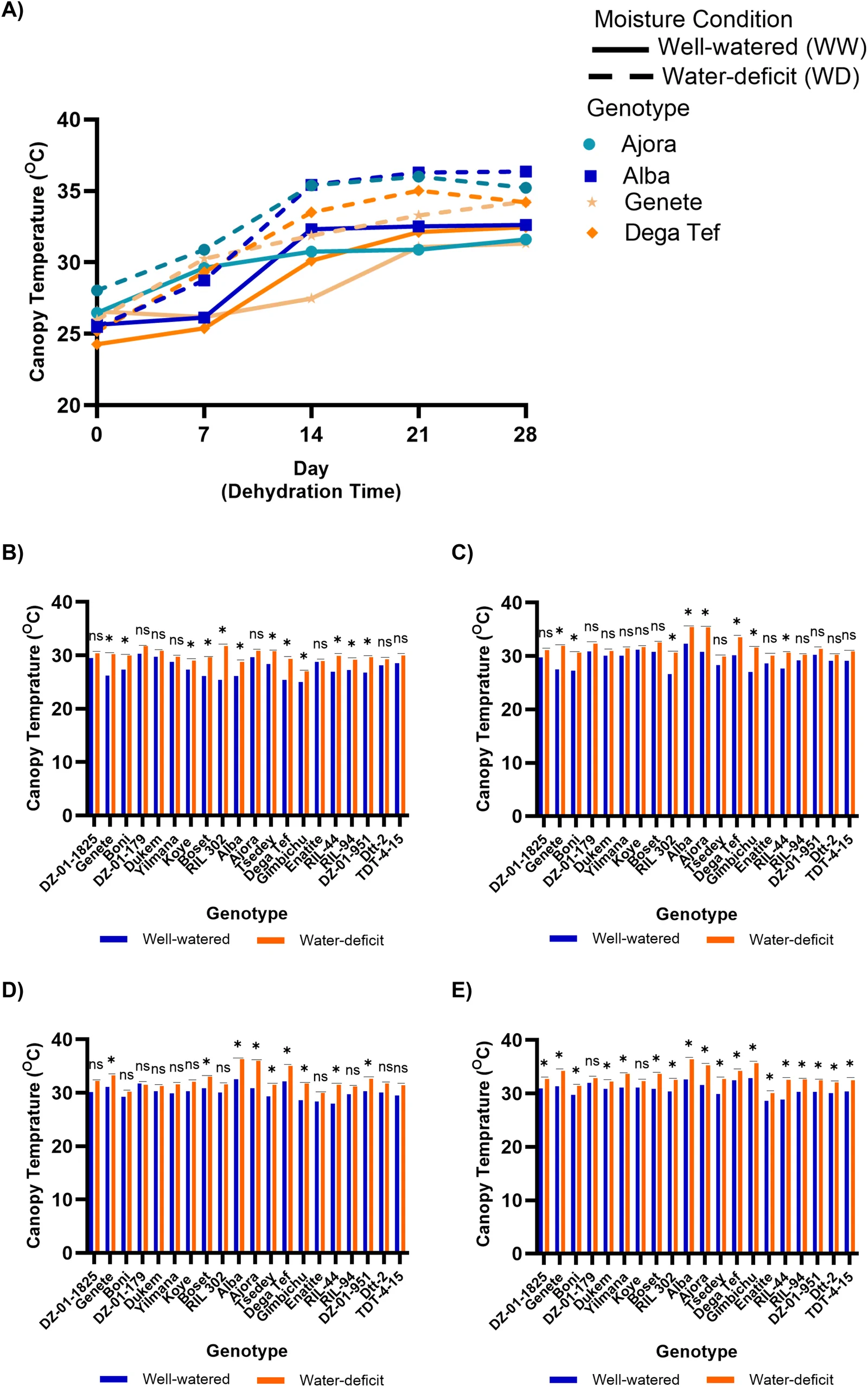
Canopy temperature (CT) for tef genotypes under well-watered (WW) and water-deficit (WD) conditions. **(A)** for four selected genotypes across four weeks; **(B)** on day 7; **(C)** Day 14; **(D)** Day 21; and **(E)** Day 28 after beginning of drought stress. Mean comparisons in panels **(B–E)** were conducted for individual genotypes between WW and WD conditions; where: *significant difference at P<0.05 and ns, non-significant.

During the first week of moisture stress, changes in NDVI, CC, or CT were not significant (P<0.05) for a lot of genotypes (**Figures 2**–**4**). However, as drought stress intensified, significant changes were observed in all physiological traits. During the first two weeks, genotypes such as RIL-302, and RIL-44 showed no significant changes in NDVI. Furthermore, Dtt-2 and TDT-4–15 exhibited no significant differences during the first week of drought stress for all three physiological traits. By the end of the fourth week, differences between WW and WD conditions were significant for most genotypes for NDVI, CC and CT.

The standard deviations of NDVI, CC (SPAD value), and CT were calculated for the 20 tef genotypes under WD conditions using measurements collected over four weeks of drought stress, beginning at drought initiation (Day 0), to assess the temporal stability of these traits during drought.

Across the four weeks of drought, RIL 44, Tsedey, RIL-302, RIL-94, Enatite, and Boni showed relatively low variation in NDVI (**Supplementary Table S7**), whereas Dukem, Enatite, RIL-302, RIL-94 and Dtt-2 exhibited minimal variation in CC (SPAD value) (**Supplementary Table S8**). In addition, genotypes such as Enatite, Dukem, DZ-01-1825, TDT-4-15, Yilmana, Koye and Dtt-2 (**Supplementary Table S9**) showed relatively low variation in CT. These findings indicate that Enatite exhibited relatively stable performance in the three physiological parameters across the four weeks of drought stress, while RIL-302 showed relatively higher stability for both CC (SPAD value) and NDVI.

Gas exchange traits under WD conditions. Significant differences were observed among tef genotypes for gs under WD conditions, suggesting differential stomatal responses of tef genotypes to changing moisture conditions (P < 0.05; **Table 3**). The lowest gs values were recorded in tef genotypes such as DZ-01-951, Alba, and RIL-44.

**Table 3:** Gas exchange traits for tef genotypes in water-deficit conditions.

Genotype	gs (mol H_2_O m^-2^ s^-1^)	A (μmol CO_2_ m^-2^ s^-1^)	E (mmol H_2_O m^-2^ s^-1^)	WUE
DZ-01-1825	54.67^a-d^	6.67^f^	3.35^de^	2.03^efgh^
Genete	56.67^a-c^	11.25^c^	4.23^bc^	2.68^cde^
Boni	33.67^c-e^	8.53^de^	2.46^f-i^	3.50^b^
DZ-01-179	56.67^a-c^	7.37^ef^	4.32^bc^	1.71^fghi^
Dukem	68.33^a^	9.57^d^	3.09^d-h^	3.10^bcd^
Yilmana	61.00^ab^	8.50^de^	3.52^cd^	2.43^def^
Koye	65.00^ab^	11.53^c^	5.20^a^	2.28^efg^
Boset	42.33^b-e^	7.37^ef^	2.29^hi^	3.25^bc^
RIL-302	50.33^a-e^	7.40^ef^	2.16^i^	3.42^bc^
Alba	31.67^de^	4.70^gh^	2.39^g-i^	2.01^efgh^
Ajora	45.33^a-e^	3.60^gh^	3.15^d-g^	1.15^ij^
Tsedey	45.00^b-e^	16.03^a^	3.15^d-g^	5.28^a^
Dega Tef	34.33^c-e^	3.37^h^	2.59^e-i^	1.40^hij^
Gimbichu	54.67^a-d^	8.30^de^	3.79^b-d^	2.19^efg^
Enatite	49.33^a-e^	13.80^b^	4.52^ab^	3.06^bcd^
RIL-44	32.67^de^	5.00^g^	2.09^i^	2.41^def^
RIL-94	48.00^a-e^	3.93^gh^	3.50^cd^	1.14^ij^
DZ-01-951	28.33^e^	1.67^i^	1.79^i^	0.92^j^
Dtt-2	45.00^b-e^	12.13^c^	3.28^d-f^	3.81^b^
TDT-4-15	51.33^a-e^	6.73^f^	4.27^bc^	1.60^ghij^
Mean	47.72	7.87	3.26	2.47
Max	68.33	16.03	5.20	5.28
Min	28.33	1.67	1.79	0.92
LSD (5%)	23.21	1.45	0.83	0.78

Where: gs, stomatal conductance; A, assimilation rate; E, transpiration rate; WUE, water use efficiency after 28 days drought stress; Max, maximum; Min, minimum; and LSD, least significant difference. Means for individual traits in each column designated with the same letter are not significantly different at P<0.05.

Assimilation rate, E and WUE are also important physiological parameters to consider in studying drought tolerance in plants (Grassi and Magnani, 2005; Alemu et al., 2024). As indicated in **Table 3**, the values of A, E and WUE were significantly different among tef genotypes under WD conditions (P < 0.01; **Supplementary Table S6**).

Since higher A values are considered beneficial to the plant, tef genotypes with the highest A values were Tsedey, Enatite, and Genete. On the other hand, low E values are associated with drought tolerance since plants lose little water through transpiration. Accordingly, plants with the lowest E values were DZ-01-951, RIL-44, and RIL-302 (**Table 3**). Tef genotypes with the highest WUE were Tsedey, Dtt2, and Boni. These findings show that the Tsedey variety had the highest A as well as the highest WUE under WD conditions indicating the variety’s suitability to the semi-arid regions.

### 3.3 Effects on phenological and agronomic traits

Effects of moisture regime on crop maturity. Tef genotypes under WD conditions reached physiological maturity earlier than those under WW conditions (P < 0.01), leading to approximately four days earlier maturity time of the crop (**Table 4**). Tef genotypes with remarkable reduction in DM due to WD compared to WW conditions include Boset from 101 to 92 days (8.5% compared to WW conditions), Gimbichu from 102 to 95 days (8.5%), Boni from 99 to 91 days (8.4%), and Dtt-2 from 100 to 92 days (8.3%). This shows that moisture scarcity hastens the maturity period of tef. Although genotypes such as DZ-01-951, DZ-01-94, and Yilmana reached maturity earlier than other genotypes under WW conditions, those that matured earlier under WD conditions were RIL-94, DZ-01-951, and Boni indicating that the later three genotypes better cope with moisture scarcity compared to the former three genotypes because they hasten their maturity time and set seeds at the time of moisture scarcity.

**Table 4:** Days to maturity (DM) of 20 tef genotypes under well-watered (WW) and water-deficit (WD) conditions.

Moisture	DM (No. of days)		
WW	100.76a		
WD	96.35b		
Genotype	WW	WD	% R
DZ-01-1825	101.00^ab^	101.00^ab^	0
Genete	105.33^a^	101.66^ab^	3.5
Boni	99.33^ab^	91.00^cd^	8.4
DZ-01-179	104.00^a^	101.00^ab^	2.8
Dukem	105.66^a^	102.33^ab^	3.1
Yilmana	94.66^b^	92.33^cd^	2.5
Koye	101.00^ab^	99.00^abc^	1.9
Boset	101.33^ab^	92.66^cd^	8.5
RIL-302	101.33^ab^	94.00^abcd^	7.2
Alba	101.33^ab^	103.66^a^	-2.3
Ajora	100.33^ab^	98.00^abcd^	2.3
Tsedey	101.00^ab^	96.33^abcd^	4.6
Dega Tef	101.66^ab^	100.66^ab^	0.9
Gimbichu	102.33^ab^	93.66^bcd^	8.5
Enatite	104.66^a^	99.33^abc^	5.1
RIL-44	102.66^ab^	95.66^abcd^	6.8
RIL-94	94.33^b^	89.66^d^	4.9
DZ-01-951	93.66^b^	90.66^cd^	3.2
Dtt-2	100.33^ab^	92.00^cd^	8.3
TDT-4-15	99.33^ab^	92.33^cd^	7.0
Mean	100.77	96.35	4.4
Max	105.67	103.67	
Min	93.67	89.67	

Where: DM, days to maturity; %R, percent reduction; Max, maximum; and Min, minimum. A non-parametric test (Kruskal-Wallis) was done to test difference between the moisture conditions and the genotypes in each moisture conditions as well because DM violated homogeneity of variance, and mean comparison was made by Benjamini-Hochberg method. Moisture conditions and genotype designated by the same letter in each column are not significantly different. Negative values of %R indicate a percentage increase.

*Thousands kernel weight (TKW)*. Compared to other cereals, the kernel or grain of tef is extremely small both in size and weight (Whisnant et al., 2024). The results of the current study showed that moisture scarcity caused a significant reduction (P < 0.05) in TKW (**Supplementary Table S10**), with an overall decrease of 0.04 g (**Table 5**). However, genotypes such as Boni, Yilmana and Dtt-2 showed no reduction between WW and WD conditions (**Figure 5A**).

**Table 5:** Mean comparison of agronomic traits in well-watered (WW) and water-deficit (WD) conditions.

Genotype	TKW (g)		GY (g/plant)		SB (g/plant)		RB (g/plant)	
	WW	WD	WW	WD	WW	WD	WW	WD
DZ-01-1825	0.34^a^	0.24^b^	3.45^a^	3.14^a^	20.11^a^	19.66^a^	0.74^a^	0.58^b^
Genete	0.29^a^	0.24^b^	3.05^a^	1.50^a^	21.44^a^	16.61^a^	0.75^a^	0.61^b^
Boni	0.34^a^	0.34^a^	3.50^a^	3.33^a^	15.33^a^	12.94^a^	0.38^a^	0.19^b^
DZ-01-179	0.29^a^	0.26^b^	2.19^a^	2.05^a^	20.55^a^	19.83^a^	0.52^a^	0.59^a^
Dukem	0.40^a^	0.28^b^	3.05^a^	2.33^b^	20.38^a^	17.88^a^	0.99^a^	0.63^b^
Yilmana	0.29^a^	0.29^a^	2.50^a^	1.47^a^	18.38^a^	15.16^a^	0.61^a^	0.45^b^
Koye	0.32^a^	0.31^a^	3.16^a^	2.70^b^	18.16^a^	11.88^b^	0.45^a^	0.34^a^
Boset	0.31^a^	0.29^a^	2.83^a^	1.94^b^	12.44^a^	8.72^b^	0.30^a^	0.23^a^
RIL-302	0.36^a^	0.25^b^	2.00^a^	1.22^b^	13.35^a^	10.88^b^	0.56^a^	0.19^b^
Alba	0.39^a^	0.25^b^	2.836^a^	1.21^b^	17.61^a^	16.94^a^	0.70^a^	0.53^b^
Ajora	0.34^a^	0.28^b^	2.53a	2.00^b^	20.00^a^	13.22^b^	0.41^a^	0.32^a^
Tsedey	0.28^a^	0.27^b^	2.54^a^	1.50^b^	13.27^a^	12.27^a^	0.32^a^	0.28^a^
Dega Tef	0.38^a^	0.32^b^	2.61^a^	1.44^b^	19.00^a^	13.22^b^	0.49^a^	0.25^b^
Gimbichu	0.34^a^	0.31^b^	3.21^a^	1.66^b^	19.94^a^	16.61^a^	0.76^a^	0.34^b^
Enatite	0.34^a^	0.30^b^	3.21^a^	3.11^b^	22.50^a^	19.00^a^	0.42^a^	0.42^a^
RIL-44	0.32^a^	0.29^b^	2.47^a^	2.05^a^	14.16^a^	8.88^b^	0.81^a^	0.39^b^
RIL-94	0.33^a^	0.31^a^	2.48^a^	2.00^a^	12.83^a^	11.66^b^	0.41^a^	0.21^b^
DZ-01-951	0.34^a^	0.28^b^	2.72^a^	1.38^b^	16.55^a^	16.49^a^	0.35^a^	0.41^a^
Dtt-2	0.29^a^	0.27^a^	3.61^a^	3.27^a^	17.16^a^	13.77^a^	0.35^a^	0.30^a^
TDT-4-15	0.30^a^	0.30a	4.55^a^	2.07^b^	21.72^a^	13.55^b^	0.53^a^	0.36^b^
Mean	0.33	0.28	2.92	2.07	17.75	14.46	0.54	0.38
Max	0.40	0.34	4.55	3.33	22.50	19.83	0.99	0.63
Min	0.28	0.24	2.00	1.21	12.44	8.72	0.30	0.19
LSD (5%)	0.022		0.52		3.62		0.14	

Where: TKW, thousand kernel weight; GY, grain yield; SB, shoot biomass; RB, root biomass; Max, maximum; Min, minimum; and LSD, least significant difference. Mean comparisons were conducted for individual traits under the two moisture conditions using the LSD test at P<0.05. Genotypes designated by the same letter are not significantly different in their response to changes in moisture conditions. Although the LSD comparison was performed across all genotypes within both moisture conditions, labeling was specifically applied to facilitate comparison of genotype performance between the two moisture regimes. Therefore, the reported LSD value applies to all genotypes and moisture conditions for each respective trait. Mean comparisons among genotypes within each individual moisture conditions are provided in the **Supplementary Materials** (**Supplementary Table S10**).

**Figure 5:**
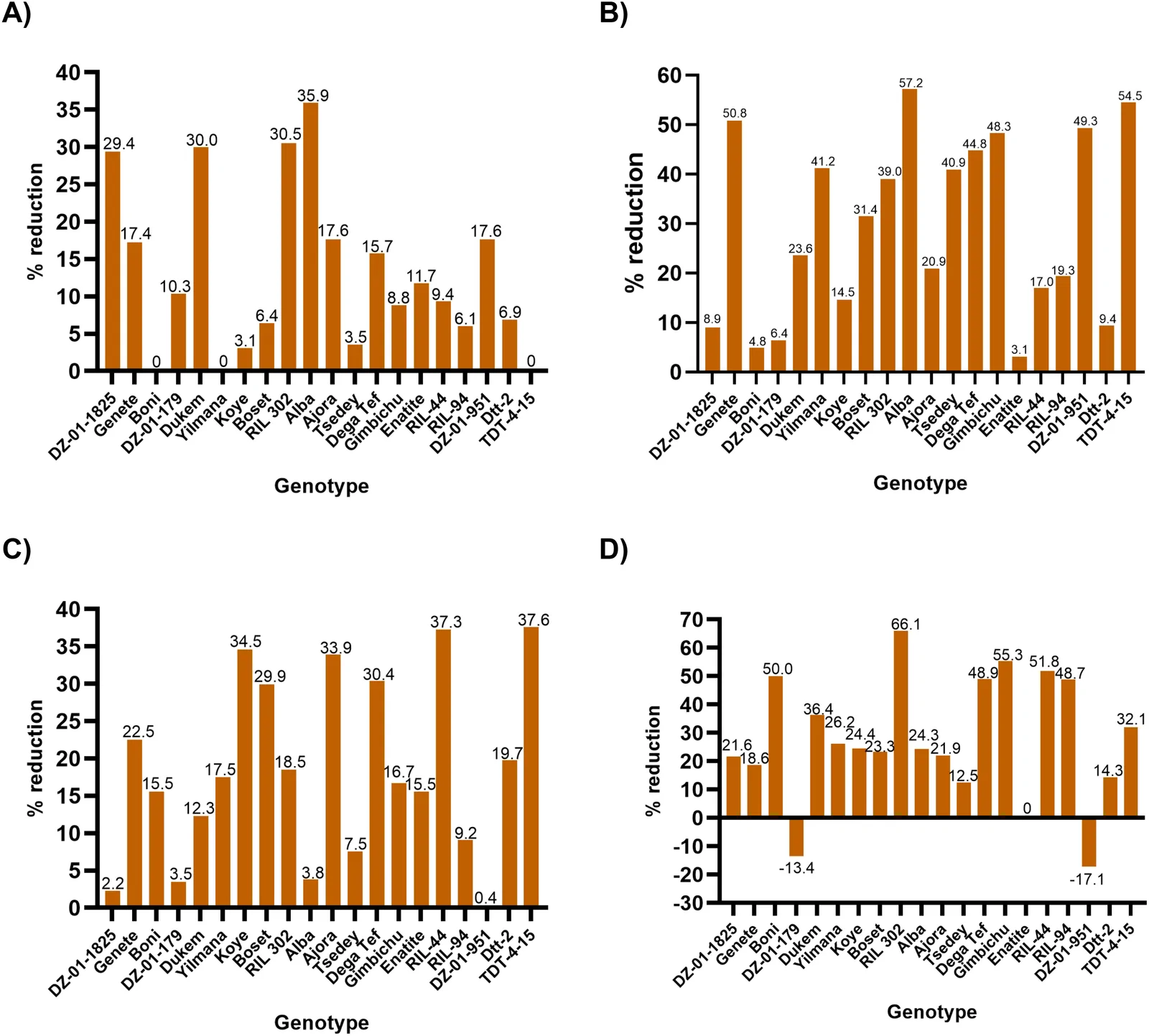
Percent reduction in selected agronomic traits in 20 tef genotypes due to moisture scarcity **(A)** Thousand kernel weight (TKW); **(B)** Grain yield (GY); **(C)** Shoot biomass (SB); and **(D)** Root biomass (RB). the number at the top each bar in the figure shows the precent reduction for each genotype. Negative values indicate percent increase.

The interaction between moisture regime and tef genotype was also significant (P < 0.001) (**Supplementary Table S6**). Significant differences among genotypes were also observed within each moisture conditions (**Supplementary Table S10**). Under WD conditions, genotypes such as Boni, Gimbichu, and Dega Tef exhibited higher TKW, whereas under WW conditions, Dukem, Alba, and Dega Tef recorded higher TKW. Interestingly, the grains of Dega Tef were heavier than majority of other tef genotypes both under WW and WD conditions indicating the maintenance of its weight even under scares moisture conditions.

*Grain yield (GY)*. Both moisture levels and tef genotypes, as well as their interaction, showed significant (P<0.001) effects on GY (**Supplementary Tables S5**, **S6**). The shift from WW to WD conditions resulted in an overall reduction in GY by 0.8g per plant (**Supplementary Table S10**). However, genotypes such as Enatite, Boni, DZ-01-179, DZ-01–1825 and Dtt-2 showed relatively little reduction between WW and WD conditions (**Figure 5B**). Except for Enatite, these genotypes showed no significant reduction in GY between the two moisture regimes (**Table 5**). Under WD conditions, genotypes with higher GY were Boni, Dtt2, DZ-01-1825, and Enatite. In contrast, under WW conditions, higher GY was recorded for TDT-4-15, Dtt2, Boni, and DZ-01-1825. Interestingly, three of the above genotypes, namely Boni, Dtt2 and DZ-01–1825 maintained their superior yield under both water regimes. This shows their suitability under high rainfall and moisture scarce environments.

*Shoot Biomass (SB)*. Both moisture levels and genotypes had significant effects (P<0.001) on SB, whereas their interaction was not significant (**Supplementary Table S6**). Under WD conditions, tef genotypes such as DZ-01-179, DZ-01-1825, and Enatite exhibited higher SB (**Supplementary Table S10**; **Figure 6**). In contrast, under WW conditions, higher SB was recorded for Enatite, TDT-4-15, and Genete. However, genotypes with small reduction in SB due to drought were DZ-01-951, DZ-01-1825, DZ-01-179, and Alba (**Figure 5C**). The reductions in SB for these genotypes, as well as for eight additional genotypes, were not significant between WW and WD conditions (**Table 5**).

**Figure 6:**
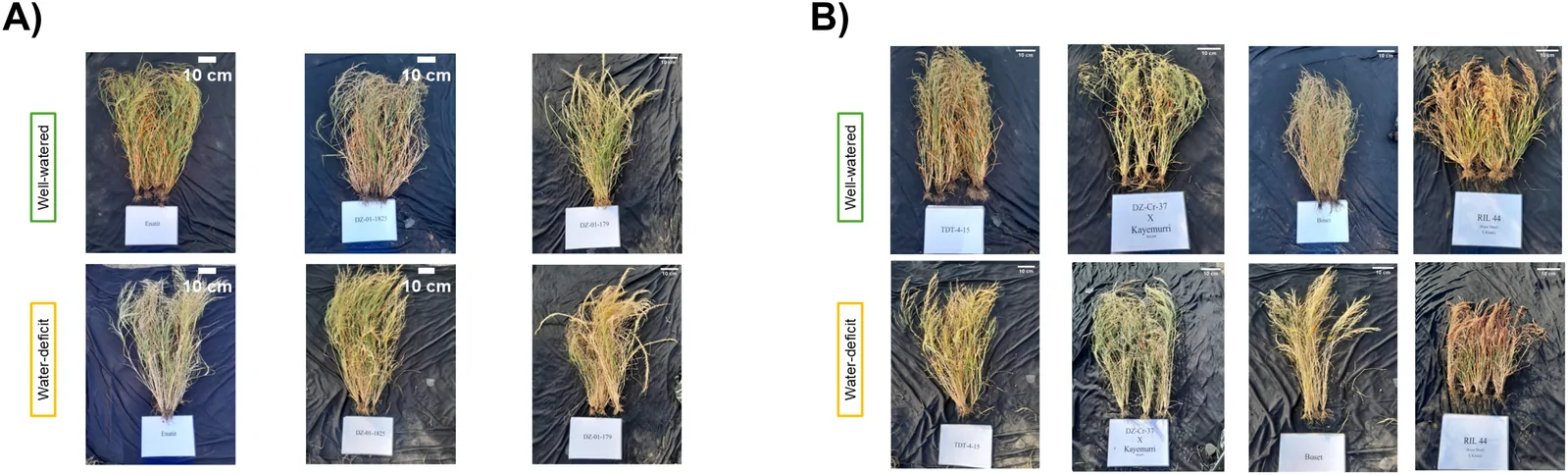
Photographs of five tef plants representing genotypes with high and low shoot biomass (SB) in well-watered (WW) and water-deficit (WD) conditions. Genotypes were selected based on their SB performance relative to the overall mean in WD conditions. **(A)** Genotypes with above-average SB in WD conditions: Enatite (left), DZ-01-1825 (middle), and DZ-01-179 (right). **(B)** Genotypes with below-average SB under WD conditions: TDT-4-15 (left), RIL-94, Boset, and RIL-44 (right).

*Root biomass (RB)*. Root biomass differed significantly (P<0.001) among moisture levels, genotypes, and their interaction (**Supplementary Tables S5**, **S6**). The change from WW to WD conditions resulted in a reduction of RB by 0.16 g per plant (**Supplementary Table S10**). Although genotypes such as DZ-01–179 and DZ-01–951 showed an increase in RB under WD condition, the differences between WW and WD were not statistically significant (**Table 5**), while Enatite showed no difference between the two contrasting moisture regimes (**Figure 5D**). Under WD conditions, higher RB was observed in genotypes such as Dukem, Genete, and DZ-01-1825. In contrast, under WW conditions, Dukem, RIL-44, and Genete recorded higher RB values. This shows that the RB of Dukem and Genete varieties was among the highest under both WW and WD conditions.

### 3.4 Multiple correlation between agronomic and physiological parameters

Chlorophyll content (SPAD value) showed a positive and significant correlation (P < 0.05) with GY, SB, TKW and NDVI when considering both moisture conditions together (**Figure 7**). Canopy temperature exhibited a negative and significant (P< 0.05) correlation with GY, NDVI, and CC (SPAD value); under WD conditions, this negative correlation was significant only for GY.

**Figure 7:**
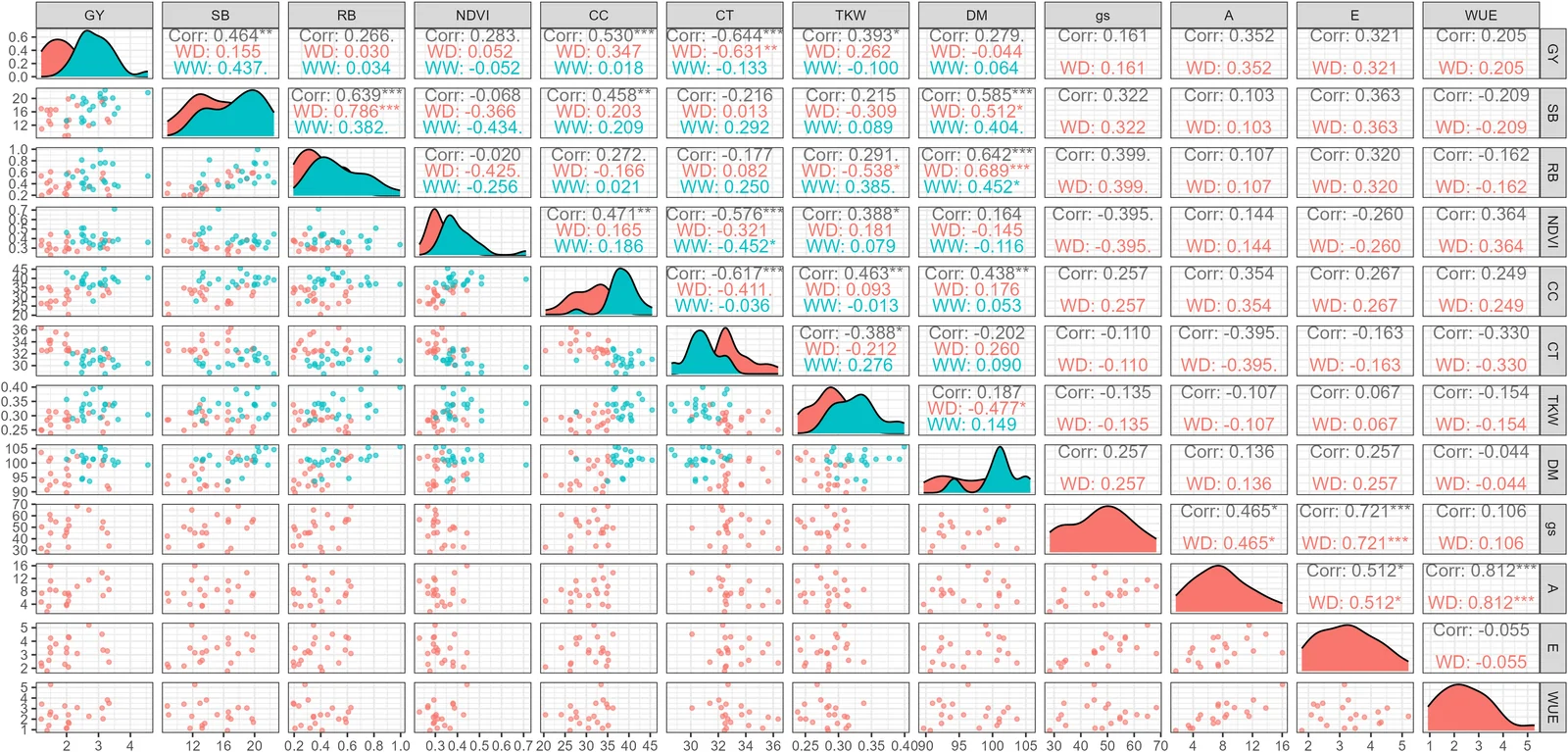
Correlation matrix 20 tef genotypes grown under well-watered (WW) and water-deficit (WD) conditions. In the upper panel, red and green text shows the correlation matrix in WW and WD conditions respectively while the black text indicates the correlation matrix of the combined data of the two conditions. The diagonal panel indicates the distribution of each trait in two moisture conditions. The lower panel shows the distribution between pairs of traits using bivariate scatter plots. *, ** and *** indicate significant at p < 0.05, p < 0.01, and p < 0. 001.Where: GY, grain yield (g plant-1); SB, shoot biomass (g plant-1); RB, root biomass (g plant-1); NDVI, normalized difference vegetation index; CC, chlorophyll content (SPAD value); CT, canopy temperature (°C); TKW, thousand kernel weight (g); DM, days to maturity; gs, stomatal conductance (mol H_2_O m -2 S-1); A, assimilation rate (μmol CO_2_ m-2 s-1); E, transpiration rate (mmol H_2_O m -2 s-1); and WUE, water use efficiency.

Thousand kernel weight showed positive and significant correlations with GY, NDVI, and CC (SPAD value), and a negative correlation (P<0.05) with CT when both moisture conditions are considered together. In WD conditions, TKW was negatively and significantly correlated with RB and DM. Root biomass showed a positive and significant correlation (P < 0.05) with SB under WD conditions. In addition, RB was positively and significantly correlated with DM under both WD and WW conditions. Furthermore, in WD conditions, the gas exchange traits such as gs, A, and E were positively and significantly correlated with one another. Water use efficiency was also positively and significantly correlated with both gs and A.

### 3.5 Principal component analysis and clustering

Principal component analysis (PCA) was used to analyze individual physiological and agronomic traits under WD conditions, assess their relationships and the observed phenotypic variation among tef genotypes, and cluster the genotypes based on their similarities.

Principal component analysis revealed substantial phenotypic variation among tef genotypes, with PC1 and PC2 explaining 31.9% and 23.7% of the total variation, respectively (**Figure 8A**). The PCA biplot indicated that WUE, A, and CC were closely associated with GY, suggesting that these traits contributed positively to GY under WD conditions. In addition, the NDVI and TKW were correlated with GY. In contrast, gs and E were closely associated with each other but were positioned separately from NDVI and TKW, indicating a distinct physiological response. Similarly, SB and RB were closely associated, suggesting that genotypes with greater RB may also tend to produce greater SB. At the same time these two agronomic traits closely associated with gs and E which could show higher RB may contribute to increase in gs and E.

**Figure 8:**
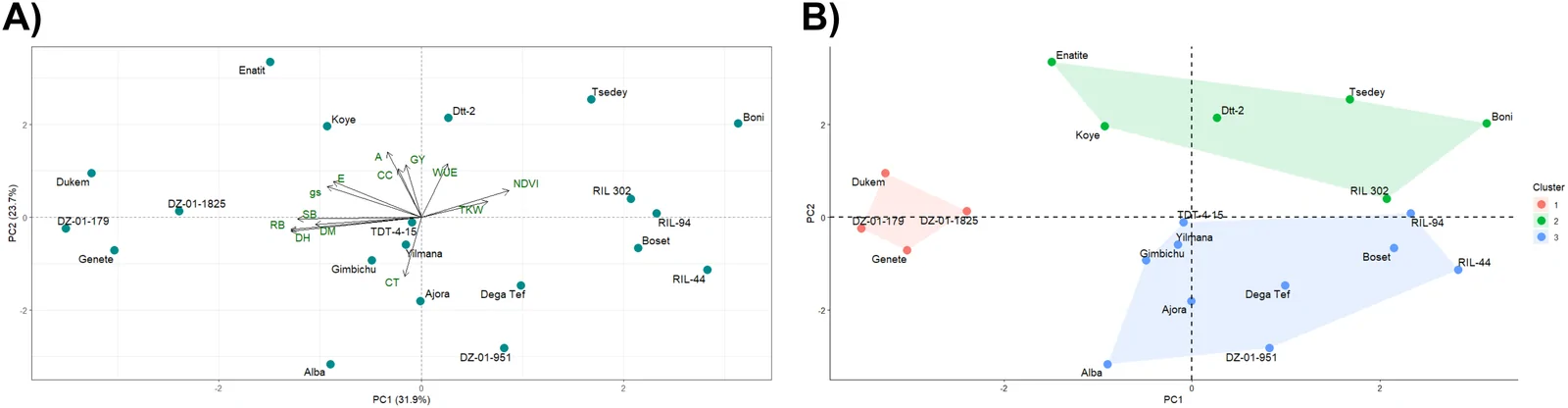
Principle component analysis (PCA) biplot of agronomic and physiological traits among 20 tef genotypes. **(A)** PCA showing the distribution of genotypes and trait contributions; and **(B)** clustering pattern of genotypes based on PCA scores. Genotypes are distributed across different quadrants according to their dissimilarity. The length of the trait vectors indicates the magnitude and quality of trait representation in the principal components, while the angles between vectors indicate the correlation patterns among traits, with acute and obtuse angles representing positive and negative associations, respectively.

Cluster analysis using PCA scores grouped the genotypes into three major clusters (**Figure 8B**), reflecting differences in agronomic performance and physiological adaptation. Cluster I comprised mainly genotypes with negative PC1 scores, characterized by longer maturity duration, higher SB, RB, gs, and E. Interestingly, these genotypes also exhibited higher CT, despite their greater transpiration capacity, suggesting that factors other than transpiration alone, such as canopy architecture, biomass accumulation, or WUE, may have influenced canopy cooling.

Cluster II consisted genotypes including Boni which is early maturing. The genotypes in this cluster showed relatively higher GY, TKW, A, and NDVI. These traits suggest enhanced photosynthetic capacity and WUE, contributing to their adaptation to semi-arid environments. Although these genotypes did not consistently exhibit the highest SB and RB, their superior GY indicates more efficient allocation of assimilated resources toward reproductive sinks compared with genotypes in Cluster I. However, it should be noted that genotype for e.g Enatite with both higher GY and SB were also grouped within Cluster II.

Cluster III included genotypes with generally lower values for most agronomic and physiological traits. Although their RB, SB, and CT were comparable to those of the more drought-adapted clusters, their lower overall trait performance suggests limited capacity for maintaining productivity under drought conditions.

### 3.6 Overall performance of tef genotype

Based on the physiological and agronomic traits evaluated, the 20 tef genotypes were classified as “+” when they showed above-average performance and “–” when they showed below-average performance, considering their performance under WD conditions and their percentage reduction (%R) between WW and WD conditions (**Table 6**). Genotypes such as Boni, Dukum, Enatite, Tsedey, and Dtt-2 showed above-average performance under WD conditions, whereas Enatite, DZ-01-179, Boni, RIL-94, and Tsedey showed above-average performance in terms of %R for most traits. Overall, Boni, Enatite, and Tsedey were identified as the best-performing candidates.

**Table 6:** Overall performance of 20 tef genotypes for physiological and agronomic traits under water-deficit (WD) conditions, based on their performance and percentage reduction (%R) relative to well-watered (WW) conditions, categorized as below (-) or above (+) the mean.

Genotype		NDVI	CC	CT	gs	A	E	WUE	DM	TKW	GY	SB	RB	NP/T
DZ-01-1825	WD	–	+	+	–	–	–	–	–	–	+	+	+	5/12
	%R	+	–	+					–	–	+	+	+	5/8
Genete	WD	–	–	–	–	+	–	+	–	–	–	+	+	4/12
	%R	–	–	–					–	–	–	–	+	1/8
Boni	WD	+	+	+	+	+	+	+	+	+	+	–	–	10/12
	%R	–	+	+					+	+	+	+	–	6/8
DZ-01-179	WD	–	+	+	–	–	–	–	–	–	–	+	+	4/12
	%R	+	+	+					–	+	+	+	+	7/8
Dukem	WD	–	+	+	–	+	+	+	–	+	+	+	+	9/12
	%R	+	+	+					–	–	+	+	–	5/8
Yilmana	WD	–	–	–	–	+	–	–	+	+	–	+	+	5/12
	%R	+	–	–					–	+	–	+	+	4/8
Koye	WD	–	+	+	–	+	–	–	–	+	+	–	–	5/12
	%R	+	–	+					–	+	+	–	+	5/8
Boset	WD	–	–	–	+	–	+	+	+	+	–	–	–	5/12
	%R	+	+	–					+	+	–	–	+	5/8
RIL-302	WD	+	+	+	–	–	+	+	+	–	–	–	–	6/12
	%R	+	+	+					+	–	–	–	–	4/8
Alba	WD	+	–	–	+	–	+	–	–	–	–	+	+	5/12
	%R	–	–	–					–	–	–	+	+	2/8
Ajora	WD	–	+	–	+	–	+	–	–	+	–	–	–	4/12
	%R	–	+	–					–	–	+	–	+	3/8
Tsedey	WD	+	+	+	+	+	+	+	+	–	–	–	–	8/12
	%R	+	+	–					+	+	–	+	+	6/8
Dega Tef	WD	+	+	–	+	–	+	–	–	+	–	–	–	5/12
	%R	+	+	+					–	–	–	–	–	3/8
Gimbichu	WD	–	–	–	–	+	–	–	+	+	–	+	–	4/12
	%R	–	–	–					–	+	–	+	–	2/8
Enatite	WD	+	+	+	–	+	–	+	–	+	+	+	+	9/12
	%R	+	+	+					+	+	+	+	+	8/8
RIL-44	WD	+	–	+	+	–	+	–	+	+	–	–	–	6/12
	%R	+	–	–					+	+	+	–	–	4/8
RIL-94	WD	+	+	+	–	–	–	–	+	+	–	–	–	5/12
	%R	+	+	–					+	+	+	+	–	6/8
DZ-01-951	WD	–	–	+	+	–	+	–	+	+	–	+	+	5/12
	%R	+	–	+					–	–	–	+	+	4/8
Dtt-2	WD	–	+	+	+	+	–	+	+	–	+	–	–	7/12
	%R	–	+	+					+	+	+	–	+	5/8
TDT-4-15	WD	–	–	+	–	–	–	–	+	+	+	–	–	4/12
	%R	+	–	+					+	+	–	–	–	4/8

Where: NDVI, normalized difference vegetation index; CC, chlorophyll content (SPAD value); CT, canopy temperature; gs, stomatal conductance; A, assimilation rate; E, transpiration rate; WUE, water use efficiency; DM, days to maturity; TKW, thousand kernel weight; GY, grain yield; SB, shoot biomass; RB, root biomass; NP, total number of positive value for a given genotypes; and NT, total number of traits. A “+” indicates that a genotype has a value above the mean for a given trait. However, for CT, E, gs, and DM, lower values are considered desirable; therefore, a “+” is assigned to genotypes with values below the mean for these traits. Conversely, a “–” indicates a value below the mean for all other traits, except CT, E, gs, and DM. The NP and NT values at the end of column indicate the overall performance of an individual genotype based on the number of favorable traits.

In contrast, Genete, DZ-01-179, Ajora, Gimbichu, and TDT-4–15 showed below-average performance under WD conditions. Similarly, Genete, Alba, Dega Tef, and Gimbichu showed below-average performance in terms of %R for most traits.

## 4 Discussion

Tef’s productivity is strongly constrained by drought, particularly when stress occurs at the reproductive stage. In this study, imposing water deficit at heading resulted in substantial reductions in plant water status, which translated into coordinated changes in physiological, phenological, and agronomic traits. These findings align with previous reports showing that terminal drought is especially detrimental to GY in tef due to impacts on flowering, fertilization, and grain filling (Admas and Belay, 2011; Shiferaw et al., 2012a; Ferede et al., 2018).

### 4.1 Plant water status and drought intensity

The imposed drought treatment significantly reduced both soil and leaf water content, confirming effective stress imposition. Similar declines in plant water status under drought have been widely reported in tef and other cereals (Degu et al., 2008; Abid et al., 2016; Qiao et al., 2024). Although tef can tolerate moderate dehydration, the observed reduction in RWC to ~52% indicates substantial physiological limitation. Previous work suggests that further reductions below this threshold can lead to loss of cellular integrity and photosynthetic function (Ginbot and Farrant, 2011; Kamies et al., 2017), supporting the severity of stress imposed in this study.

### 4.2 Physiological responses underpin drought tolerance

Spectral based physiological measurements have been found to be quick indicators of plant stress and have been used to indicate traits associated with drought tolerant genotypes (Wen et al., 2023). Normalized difference vegetation index is a spectral based tool that is commonly used as an indicator of canopy greenness and vegetation density, with higher values reflecting greater photosynthetic activity (Gamon et al., 1995; Ding et al., 2022). On the other hand, hand-held spectral absorbance device (SPAD) is used to estimate the total CC. Leaf CC is a key determinant of photosynthetic potential and is strongly influenced by environmental stresses such as drought (Zhang et al., 2011; Alemu et al., 2024). Higher CC values generally indicate healthier, more photosynthetically active plants and are positively associated with biomass accumulation and GY (Castelli and Contillo, 2009). The CT of the plant is key indicator of the water status and plays vital role in physiological processes such as photosynthesis, respiration, and transpiration (Takele, 2001).

Drought stress reduced NDVI and CC (SPAD value) while increasing CT, consistent with findings in wheat, sorghum, and other crops (Ram et al., 2017; Ding et al., 2022; Vijayalakshmi et al., 2024). These changes reflect declining photosynthetic capacity, chlorophyll degradation, and reduced canopy function under water limitation (Monteoliva et al., 2021).

However, strong genotypic variation was evident. Genotypes such as Enatite, Tsedey, RIL-44, and RIL-94, maintained smaller reduction and higher NDVI values in WD conditions, while Enatite, and Dukem retained higher CC (SPAD value) with relatively smaller reductions compared with WW conditions. Maintenance of these traits under drought is often associated with sustained photosynthesis and yield stability under stress (Peters et al., 2002; Wen et al., 2023). In crops such as sorghum, this trait is related to “stay-green” phenotype (Monteoliva et al., 2021). Although a well-defined stay-green phenotype has not been reported in tef, genotypes with higher and sustained NDVI and CC (SPAD value) over the duration of drought in this study may exhibit functionally similar characteristics. Positive associations between CC (SPAD value), and GY, as well as between NDVI and TKW observed here, are consistent with previous studies in wheat and other cereals (Ram et al., 2017; Kizilgeci et al., 2021).

Canopy temperature also proved informative, with lower values and smaller increase in WD conditions compared to WW conditions in genotypes such as Enatite, Boni, and Dtt-2 indicating improved plant water status. Lower CT under drought has been linked to enhanced transpiration efficiency and improved yield in wheat and rice (Abdelhakim et al., 2021; Fukai and Mitchell, 2022). The negative relationship between CT and yield observed here is similarly well documented (Sareen et al., 2023).

Gas exchange responses revealed contrasting drought tolerance strategies. Genotypes such as Dukem maintained relatively higher stomatal conductance and transpiration, likely supported by improved water uptake, as also reported for tef genotypes with larger root systems (Degu et al., 2008). In contrast, genotypes such as Boni and Dtt-2 exhibited higher WUE through reduced or intermediate gs while maintaining A. This balance between carbon gain and water loss is recognised as a key indicator of drought tolerance (Grassi and Magnani, 2005; Alemu et al., 2024).

These findings support the concept that regulated gas exchange, rather than maximal conservation or maximal assimilation, underpins improved performance under drought, consistent with observations in wheat and other cereals (Sareen et al., 2023; Alemu et al., 2024).

### 4.3 Agronomic consequences and yield stability

Drought significantly reduced GY, SB, TKW, and RB, consistent with previous reports in tef and other cereals (Abraha et al., 2017; Ferede et al., 2018; Xu et al., 2023). These reductions are largely attributable to impaired photosynthesis, reduced assimilate availability, and disruption of source–sink relationships (Li et al., 2023).

Despite these general trends, several genotypes—particularly Boni, Dtt-2, Enatite, and DZ-01-1825—maintained relatively high GY with minimal reductions under drought. Their performance reflects favourable combinations of physiological traits, including higher WUE, moderated gs, and cooler canopies. Similar trait combinations have been linked to yield stability under drought in wheat (Fang et al., 2017; Sewore et al., 2023).

Thousand kernel weight showed a positive association with GY, supporting previous findings that grain weight is a key determinant of yield stability under drought (Fang et al., 2017). The stability of both yield and kernel weight in Boni highlights the importance of selecting for stability rather than maximum yield potential alone (Simane et al., 1993).

Root biomass showed a more complex relationship with yield and yield components. While larger root systems can enhance water acquisition (Degu et al., 2008), they do not always translate into higher yield, likely due to trade-offs in carbon allocation (Ober and Heathcote, 2024). Similar inconsistencies have been reported in wheat, where excessive root growth can reduce yield under terminal drought (Figueroa-Bustos et al., 2020).

### 4.4 Drought adaptation strategies

Two primary drought-response strategies were evident:

- Drought escape, characterised by earlier maturity, reduces exposure to terminal stress (Ilyas et al., 2021). This was evident in genotypes such as Boni and aligns with previous findings in tef (Abraha, 2016; Ferede et al., 2020).
- Drought avoidance, involving reduced water loss or enhanced water uptake, was observed in genotypes such as Dukem and Genete and has been widely reported in tef and other cereals (Degu et al., 2008; Mengistu, 2009; Fang and Xiong, 2015).

Importantly, the most productive genotypes (e.g., Boni and Dtt-2) exhibited intermediate strategies, balancing water conservation with sustained assimilation. This aligns with the isohydric–anisohydric framework, where intermediate behaviour is often associated with optimal productivity under fluctuating water availability (Alemu et al., 2024).

### 4.5 Synthesis and implications

Drought tolerance in tef emerges as a multi-trait phenomenon involving coordinated physiological and developmental responses. Key traits associated with improved performance include:

- Maintenance of NDVI and chlorophyll
- Lower canopy temperature
- Balanced gas exchange and improved water use efficiency
- Yield stability across contrasting moisture regimes

Genotypes such as Enatite, Boni, and Dtt-2 consistently combined most of these traits, making them strong candidates for breeding programmes.

## 5 Conclusion

The effect of drought at terminal growth stage were comparatively evaluated using physiological and agronomic traits by applying no further irrigation to supply for WD conditions after heading of the plants while the control regularly supplied with irrigation. Variations in studied traits were observed under optimum and drought-stressed conditions. Terminal drought stress resulted in reduced physiological and agronomic traits, increase in CT and early maturity. Balancing between A and E could enable tef genotypes to better cope with moisture deficit, lower their CT, have better growth and productivity. The high yielding characteristics of genotypes such as Boni, Dtt2, and Enatite may be related to their higher WUE under drought. This shows the plasticity of the genotype to the change in soil moisture. This study shows that drought escape, and drought avoidance are vital agronomic mechanisms in maintaining the productivity of tef plant exposed to moisture stress. Future studies should validate these findings across diverse environments and integrate physiological, root architectural, and molecular traits to give more insight into the mechanism of drought tolerance at terminal growth stage and accelerate the development of drought tolerant varieties.

## Data Availability

The datasets generated for this study and the **Supplementary Material** can be found in https://figshare.com/s/66c29114a0bc83feb7ce.

## In memoriam

We dedicate this paper to the late Dr. Kebebew Assefa, honoring his significant contributions to this work and his enduring legacy in advancing tef research and improvement.
