# Supplementary material for: The effect of drought on physiological and agronomic traits of tef [Eragrostis tef (Zucc.) Trotter]
Source: Front Plant Sci. 2026 Sep 16;17:1880325. doi: 10.3389/fpls.2026.1880325 (PMC13624852; doi:10.3389/fpls.2026.1880325)
Supplement: Supplementary file 1 [file Supplementaryfile1.docx]

***Supplementary Material***

Total No. of Pages: **12**, Total Number of Tables:**10**, Total Number of Figures: **2**

**Table S1**. Physical and chemical properties of the soil at the experimental field.

| Physical Property | | Chemical Property | |
| --- | --- | --- | --- |
| Clay (%) | 25.00 | Total Nitrogen (%) | 0.216 |
| Silt (%) | 38.33 | Phosphorus (mg/kg soil) | 68.35 |
| Sand (%) | 36.67 | Potassium (meq/100g) | 3.16 |
| Textural Class | Loam | Organic Carbon (%) | 2.72 |
|  |  | P^H^ | 6.95 |

**Table S2** Description of physiological data taken.

| Parameter | Unit | Description |
| --- | --- | --- |
| Normalize Deference Vegetation Index (NDVI) |  | Measurements were performed using a handheld portable Green Seeker. It measures reflectance to provide detailed understanding of plant health and vigor at two different weave lengths. NDVI is the ratio of the difference in reflectance between near infrared (NIR) and red lights to the sum of the two (Schumacher et al., 2009). It was measured between 10:00 a.m. and 2:00 p.m. under clear sky to avoid light fluctuation. |
| Chlorophyll Content (CC) |  | Soil and Plant Analysis Division (SPAD) values of fully expanded uppermost leaves (flag leaf) was determined. SPAD readings were taken at the mid of the leaf which 1/2 of the distance from the leaf base using handheld portable SPAD (SPAD-502, Minolta Corp.) on cloudless day between 10am and 2pm. |
| Canopy Temperature (CT): | ^O^C | This was measured using a handheld infrared thermometer positioned approximately 50 cm above the canopy at an angle of approximately 45° to the horizontal. It was also taken between 10 am to 2pm in a cloudless day. |
| Gas Exchange (A, gs, E, and WUE) |  | It was measured on flag leaves aligned to cover a 6cm^2^ (2 cm cuvette leaf length by 3 cm leaf width at the midpoint of the leaf) cuvette between 10am and 2pm under clear sky to avoid light fluctuation. These measurements were made with a portable CO2/H2O analyser (CIRAS-3, PP Systems, Inc., Amesbury, USA). Natural light was used to irradiate on to the leaf. The measurement was performed under adjusted light intensity 1200 u mol m^-2^ S^-1^, flow rate of 300 μmol CO_2_ S^-1^, and atmospheric pressure of 830 mbar. The temperature in the sample chamber was controlled at 25^O^C. Ambient CO_2_ was used. Where: gs (Stomatal Conductance): mol H₂O m⁻² s⁻¹, A (Assimilation Rate): μmol CO_2_ m⁻² s, E (Transpiration Rate): mmol H₂O m⁻² s⁻¹, and WUE (Water Use Efficiency): the ratio of A to E was taken |

**Table S3** Description of agronomic data taken at the physiological maturity.

| Parameter | Unit | Description |
| --- | --- | --- |
| Thousand Seed Weight (TKW) | gm | About 350 seeds with moisture content 12.5% were taken and TKW was calculated by Marvin ProLine II seed analyzer (MARViTECH, Germany). |
| Grian Yield (GY) | gm | The weight of grain for the plant in the plot. The GY per plant was calculated by diving GY per plot by 50 which is number of plants in each plot |
| Shoot Biomass (SB) | gm | The dry weight of straw and the chaff including tillers of the plants in the plot. The SB per plant was calculated by dividing SB per plot by 50 which is number of plants in each plot |
| Root Biomass (RB) | gm | After harvesting the above-ground biomass, roots from all individual plants within each plot were excavated using a hoe to a depth of approximately 30 cm and in a 50 cm radius around each plant, where the highest root density is typically found in many crops (Ciro et al., 2020). For both WW and WD conditions the roots were taken while the soil is dry. After that, small soil particles attached to the roots were removed, and the harvested roots were air-dried for two weeks to ensure that no moisture residue remained in the roots. After two weeks, the dried roots were weighed for each plot. The RB per plant was calculated by dividing RB per plot by 50 which is number of plants in each plot. |

**Table S4**. Physiological traits of 20 tef genotypes under well-watered (WW) and water-deficit (WD) conditions.

| Moisture | NDVI | | CC (SPAD value) | | CT (^O^C) | |
| --- | --- | --- | --- | --- | --- | --- |
| WW | 0.41a | | 38.53a | | 30.82b | |
| WD | 0.32b | | 30.53b | | 33.10a | |
| LSD (5%) | 0.01 | | 0.98 | | 0.28 | |
| Genotype | WW | WD | WW | WD | WW | WD |
| DZ-01-1825 | 0.37e-g | 0.31e-g | 40.58c | 30.60a-e | 30.97c-g | 32.69e-g |
| Genete | 0.30h | 0.23h | 37.57e-i | 27.57d-f | 31.33b-e | 34.26bc |
| Boni | 0.71a | 0.38c | 39.56cd | 32.22a-e | 29.73g-i | 31.39gh |
| DZ-01-179 | 0.36gh | 0.30fg | 40.47c | 32.60a-d | 32.00a-c | 32.90d-f |
| Dukem | 0.34gh | 0.27gh | 36.81hi | 35.03a | 30.87c-g | 32.21fg |
| Yilmana | 0.37fg | 0.30e-g | 38.49d-g | 24.1fg | 31.07c-f | 33.70c-e |
| Koye | 0.36gh | 0.30e-g | 45.44a | 31.78a-e | 31.07c-f | 32.32fg |
| Boset | 0.33gh | 0.27h | 27.68k | 26.28e-g | 30.90c-g | 33.67c-e |
| RIL-302 | 0.46c | 0.38c | 43.14b | 34.76a | 30.37d-g | 32.57e-g |
| Alba | 0.43c-f | 0.33d-f | 36.4ij | 26.39e-g | 32.63ab | 36.36a |
| Ajora | 0.43c-e | 0.31e-g | 36.90g-i | 30.51a-e | 31.6a-d | 35.23ab |
| Tsedey | 0.51b | 0.44a | 40.64c | 33.46a-d | 29.93f-h | 32.71e-g |
| Dega Tef | 0.38e-g | 0.34c-e | 42.37b | 35.13a | 32.47ab | 34.21b-d |
| Gimbichu | 0.38e-g | 0.29fg | 36.28ij | 27.69c-f | 32.83a | 35.63a |
| Enatite | 0.45cd | 0.39bc | 39.18c-e | 36.26a | 28.60i | 30.09h |
| RIL-44 | 01010 | 0.43ab | 37.03f-i | 20.40g | 28.83hi | 32.51e-g |
| RIL-94 | 0.44cd | 0.37cd | 38.64d-f | 33.80a-c | 30.33d-g | 32.59e-g |
| DZ-01-951 | 0.35gh | 0.28gh | 35.00j | 26.19e-g | 30.33d-g | 32.46e-g |
| Dtt-2 | 0.39d-g | 0.29fg | 38.13d-h | 33.94ab | 30.07e-h | 32.04fg |
| TDT-4-15 | 0.36gh | 0.29g | 40.36c | 28.41b-f | 30.40d-g | 32.50e-g |
| Mean | 0.41 | 0.32 | 38.53 | 30.36 | 30.82 | 33.10 |
| LSD (5%) | 0.06 | 0.05 | 1.62 | 6.16 | 1.31 | 1.33 |

Where: NDVI: normalized difference vegetation index, CC: chlorophyll content (SPAD value), and CT: canopy temperature. **Note:** Mean comparison was done separately for each moisture conditions and trait. Genotype designated by the same letter in each column are not significantly different at P<0.05.

**Table S5**. Individual analysis of variance of in two moisture conditions for physiological and agronomic traits.

| Well-watered (WW) | | | | | |
| --- | --- | --- | --- | --- | --- |
| Traits | Genotype | Rep | Block (Rep) | Error | CV% |
| Df | 19 | 2 | 9 | 29 |  |
| NDVI | 0.02*** | 0.01ns | 0.01ns | 0.01 | 9.03 |
| CC | 39.45*** | 1.23ns | 1.08ns | 0.94 | 2.51 |
| CT | 3.91*** | 0.08ns | 0.13ns | 0.61 | 2.53 |
| DM | 42.92** |  |  |  |  |
| TKW | 0.01*** | 0.01ns | 0.01ns | 0.01 | 4.55 |
| GY | 1.01*** | 0.17ns | 0.06ns | 0.08 | 9.68 |
| SB | 31.03** | 4.00ns | 5.29ns | 9.38 | 17.25 |
| RB | 0.11*** | 0.02ns | 0.01ns | 0.01 | 15.82 |
| Water-deficit (WD) | | | | | |
| NDVI | 0.01*** | 0.01ns | 0.01ns | 0.01 | 8.61 |
| CC | 55.34*** | 45.25ns | 37.59* | 13.62 | 12.15 |
| CT | 6.53*** | 0.73ns | 0.97ns | 0.63 | 2.40 |
| DM | 55.02*** |  |  |  |  |
| TKW | 0.01*** | 0.01ns | 0.01ns | 0.01 | 3.57 |
| GY | 1.46*** | 0.06ns | 0.14ns | 0.13 | 17.41 |
| SB | 3.32*** | 0.36ns | 1.05ns | 1.62 | 8.80 |
| RB | 0.06*** | 0.02* | 0.01ns | 0.01 | 19.06 |
| Gs | 389.27* | 1342.52** | 265.69 | 193.16 | 29.12 |
| A | 0.46** | 4.76*** | 0.45* | 0.16 | 7.12 |
| E | 41.52*** | 0.56ns | 0.91ns | 0.76 | 11.07 |
| WUE | 2.60*** | 1.86** | 0.33ns | 0.25 | 15.27 |

Where: df: degree of freedom, Rep: replications, CV: coefficient of variation, NDVI: normalized difference vegetation index, CC: chlorophyll content (SPAD value), CT: canopy temperature, DM: days to maturity, TKW: thousand kernel weight; SB: shoot biomass, RB: root biomass, gs: stomatal conductance, A: assimilation rate, E: transpiration and WUE: water use efficiency. **Note:** For DM non parameter Kruskal-Wallis chi-square test was done. Numbers designated by *, **, and *** indicate significant differences at P < 0.05, P < 0.01, and P < 0.001, respectively, while ns indicates a non-significant difference.

**Table S6**. Combined analysis of variance for 20 genotypes in two moisture conditions for physiological, yield and yield related traits.

| SOV | G | M | G*M | R |  | B(R) | | B(R)*M | | Error | | CV% | |
| --- | --- | --- | --- | --- | --- | --- | --- | --- | --- | --- | --- | --- | --- |
| Df | 19.00 | 1.00 | 19.00 | 2.00 | 9.00 | | 11.00 | | 58.00 | |  | |  |
| NDVI | 0.038*** | 0.236*** | 0.005*** | 0.002ns | 0.00089ns | | 0.002ns | | 0.001 | | 24.60 | |  |
| CC | 70.10*** | 20006.40*** | 24.70*** | 17.30ns | 21.50** | | 19.40** | | 7.3 | | 18.18 | |  |
| CT | 9.43*** | 156.62*** | 1.01ns | 0.35ns | 0.40ns | | 0.66ns | | 0.62 | | 5.75 | |  |
| TKW | 0.003*** | 0.062*** | 0.002*** | 0.00001ns | 0.00008ns | | 0.0003ns | | 0.0002 | | 4.56 | |  |
| GY | 1.89*** | 21.92*** | 0.57*** | 0.17ns | 0.13ns | | 0.07ns | | 0.11 | | 13.26 | |  |
| SB | 56.30*** | 323.30*** | 8.00ns | 3.10ns | 4.20ns | | 2.00ns | | 5.50 | | 14.56 | |  |
| RB | 0.14*** | 0.75*** | 0.03*** | 0.01ns | 0.01ns | | 0.02** | | 0.01 | | 21.27 | |  |

Where: G: genotype, M: moisture, R: replication, B: block, CV: coefficient of variation, NDVI: normalized difference vegetation index, CC: chlorophyll content (SPAD value), CT: canopy temperature, TKW: thousand kernel weight, GY: grain yield, SB: shoot biomass, and RB: root biomass. **Note:** Sources of variation (G, M, and others) are presented in the rows, with their corresponding degrees of freedom (df) shown below. The physiological and agronomic traits are listed in the first column. Numbers designated by *, **, and *** indicate significant differences at P < 0.05, P < 0.01, and P < 0.001, respectively, while ns indicates a non-significant difference.

**Table S7**. Changes in the normalized difference vegetation index (NDVI) from the onset of drought (Day 0) to 28 days after drought initiation (Day 28) under water-deficit (WD) conditions for 20 tef genotypes.

| Genotype | NDVI | | | | | STDV |
| --- | --- | --- | --- | --- | --- | --- |
|  | Day 0 | Day 7 | Day 14 | Day 21 | Day 28 |  |
| DZ-01-1825 | 0.67 | 0.67 | 0.56 | 0.48 | 0.31 | 0.13 |
| Genete | 0.67 | 0.56 | 0.45 | 0.30 | 0.23 | 0.16 |
| Boni | 0.62 | 0.67 | 0.65 | 0.54 | 0.38 | 0.11 |
| DZ-01-179 | 0.71 | 0.60 | 0.45 | 0.37 | 0.30 | 0.15 |
| Dukem | 0.67 | 0.60 | 0.47 | 0.34 | 0.27 | 0.15 |
| Yilmana | 0.73 | 0.67 | 0.55 | 0.40 | 0.30 | 0.16 |
| Koye | 0.69 | 0.65 | 0.53 | 0.41 | 0.30 | 0.15 |
| Boset | 0.61 | 0.62 | 0.56 | 0.39 | 0.27 | 0.14 |
| RIL 302 | 0.53 | 0.59 | 0.61 | 0.52 | 0.38 | 0.08 |
| Alba | 0.70 | 0.68 | 0.57 | 0.46 | 0.33 | 0.14 |
| Ajora | 0.68 | 0.64 | 0.54 | 0.44 | 0.31 | 0.14 |
| Tsedey | 0.58 | 0.62 | 0.70 | 0.60 | 0.44 | 0.08 |
| Dega Tef | 0.69 | 0.69 | 0.56 | 0.45 | 0.34 | 0.13 |
| Gimbichu | 0.62 | 0.66 | 0.55 | 0.39 | 0.29 | 0.14 |
| Enatit | 0.68 | 0.65 | 0.59 | 0.46 | 0.39 | 0.11 |
| RIL-44 | 0.50 | 0.59 | 0.63 | 0.54 | 0.43 | 0.07 |
| RIL-94 | 0.56 | 0.64 | 0.62 | 0.53 | 0.37 | 0.10 |
| DZ-01-951 | 0.72 | 0.68 | 0.61 | 0.43 | 0.28 | 0.17 |
| Dtt-2 | 0.65 | 0.67 | 0.60 | 0.45 | 0.29 | 0.14 |
| TDT-4-15 | 0.64 | 0.67 | 0.53 | 0.41 | 0.29 | 0.14 |

Where: STDV: standard deviation. **Note**: NDVI values and their standard deviations are presented to assess the stability of genotypic performance over the four-weeks of drought period.

**Table S8**. Changes in chlorophyll content (CC; SPAD value) from the onset of drought (Day 0) to 28 days after drought initiation (Day 28) under water-deficit (WD) conditions for 20 tef genotypes.

| Genotype | CC (SPAD value) | | | | | STDV |
| --- | --- | --- | --- | --- | --- | --- |
|  | Day 0 | Day 7 | Day 14 | Day 21 | Day 28 |  |
| DZ-01-1825 | 37.00 | 42.89 | 36.01 | 30.60 | 30.60 | 4.5 |
| Genete | 44.11 | 38.82 | 38.98 | 27.57 | 27.57 | 6.6 |
| Boni | 42.00 | 39.58 | 37.62 | 34.20 | 32.22 | 3.5 |
| DZ-01-179 | 44.73 | 38.59 | 33.09 | 34.72 | 32.60 | 4.5 |
| Dukem | 38.59 | 38.04 | 36.59 | 36.38 | 35.03 | 1.3 |
| Yilmana | 35.74 | 43.13 | 35.22 | 24.10 | 24.10 | 7.4 |
| Koye | 40.40 | 39.67 | 36.53 | 35.11 | 31.78 | 3.1 |
| Boset | 41.73 | 42.08 | 33.74 | 31.63 | 26.28 | 6.1 |
| RIL 302 | 43.03 | 38.67 | 40.23 | 37.42 | 34.76 | 2.7 |
| Alba | 38.49 | 40.26 | 37.74 | 37.50 | 26.39 | 4.9 |
| Ajora | 38.29 | 39.01 | 32.57 | 31.62 | 30.51 | 3.5 |
| Tsedey | 41.80 | 42.22 | 41.60 | 36.79 | 33.46 | 3.5 |
| Dega Tef | 38.31 | 43.87 | 42.93 | 35.13 | 35.13 | 3.7 |
| Gimbichu | 38.23 | 41.01 | 37.37 | 27.69 | 27.69 | 5.6 |
| Enatit | 43.07 | 37.54 | 38.69 | 35.34 | 36.26 | 2.6 |
| RIL-44 | 43.14 | 42.51 | 40.96 | 35.48 | 20.40 | 8.5 |
| RIL-94 | 39.44 | 39.84 | 42.93 | 33.52 | 33.80 | 3.6 |
| DZ-01-951 | 36.40 | 40.81 | 34.28 | 29.79 | 26.19 | 5.1 |
| Dtt-2 | 39.22 | 39.67 | 32.38 | 35.06 | 33.94 | 2.9 |
| TDT-4-15 | 39.11 | 39.92 | 37.01 | 31.74 | 28.41 | 4.4 |

Where: STDV: standard deviation. **Note**: CC (SPAD value) and their standard deviations are presented to assess the stability of genotypic performance over the four-weeks of drought period.

**Table S9**. Changes in canopy temperature (CT) from the onset of drought (Day 0) to 28 days after drought initiation (Day 28) under water-deficit (WD) conditions for 20 tef genotypes.

| Genotype | CT (^O^C) | | | | | STDV |
| --- | --- | --- | --- | --- | --- | --- |
|  | Day 0 | Day 7 | Day 14 | Day 21 | Day 28 |  |
| DZ-01-1825 | 28.57 | 30.41 | 31.08 | 32.23 | 32.69 | 1.46 |
| Genete | 25.98 | 30.26 | 31.88 | 33.30 | 34.26 | 2.91 |
| Boni | 23.94 | 29.98 | 30.59 | 30.20 | 31.39 | 2.68 |
| DZ-01-179 | 25.48 | 31.77 | 32.31 | 31.50 | 32.90 | 2.70 |
| Dukem | 28.40 | 30.84 | 30.96 | 31.27 | 32.21 | 1.26 |
| Yilmana | 28.82 | 29.71 | 31.42 | 31.60 | 33.70 | 1.69 |
| Koye | 27.90 | 29.03 | 31.63 | 32.10 | 32.32 | 1.79 |
| Boset | 26.74 | 29.63 | 32.54 | 33.00 | 33.67 | 2.58 |
| RIL 302 | 25.60 | 31.74 | 30.63 | 31.57 | 32.57 | 2.49 |
| Alba | 25.49 | 28.74 | 35.42 | 36.30 | 36.36 | 4.50 |
| Ajora | 28.04 | 30.89 | 35.39 | 36.00 | 35.23 | 3.12 |
| Tsedey | 24.21 | 30.74 | 29.90 | 31.53 | 32.71 | 2.95 |
| Dega Tef | 25.19 | 29.33 | 33.51 | 35.03 | 34.21 | 3.70 |
| Gimbichu | 24.02 | 27.00 | 31.59 | 31.77 | 35.63 | 4.05 |
| Enatite | 28.79 | 28.93 | 30.09 | 30.00 | 30.09 | 0.59 |
| RIL-44 | 25.78 | 29.88 | 30.63 | 31.53 | 32.51 | 2.32 |
| RIL-94 | 23.70 | 29.18 | 30.26 | 31.20 | 32.59 | 3.06 |
| DZ-01-951 | 26.51 | 29.62 | 31.34 | 32.60 | 32.46 | 2.26 |
| Dtt-2 | 26.64 | 29.23 | 30.24 | 31.73 | 32.04 | 1.95 |
| TDT-4-15 | 27.72 | 29.94 | 30.86 | 31.40 | 32.50 | 1.61 |

Where: STDV: standard deviation. **Note**: CT values and their standard deviations are presented to assess the stability of genotypic performance over the four-weeks of drought period.

**Table S10**. Agronomic traits in well-watered (WW) and water-deficit (WD) conditions.

| Moisture | TKW (g) | | GY (g/plant) | | SB (g/plant) | | RB (g/plant) | |
| --- | --- | --- | --- | --- | --- | --- | --- | --- |
| WW | 0.32a | | 2.92a | | 17.74a | | 0.54a | |
| WD | 0.28b | | 2.07b | | 14.46b | | 0.38b | |
| LSD (5%) | 0.005 | | 0.12 | | 0.85 | | 0.02 | |
| Genotype | WW | WD | WW | WD | WW | WD | WW | WD |
| DZ-01-1825 | 0.34c-e | 0.24j | 3.45bc | 3.14ab | 20.11a-d | 19.66a | 0.74bc | 0.58ab |
| Genete | 0.29h-k | 0.24j | 3.05c-e | 1.50e-i | 21.44a-c | 16.61cd | 0.75b | 0.61ab |
| Boni | 0.34c-f | 0.34a | 3.50bc | 3.33a | 15.33d-h | 12.94fg | 0.38h-k | 0.19k |
| DZ-01-179 | 0.29h-k | 0.26hi | 2.19gh | 2.05d-f | 20.55a-c | 19.83a | 0.52e-h | 0.59ab |
| Dukem | 0.40a | 0.28e-g | 3.05c-e | 2.33cd | 20.38a-d | 17.88a-c | 0.99a | 0.63a |
| Yilmana | 0.29h-k | 0.29de | 2.50fg | 1.47f-i | 18.38a-f | 15.16de | 0.61c-e | 0.45c-e |
| Koye | 0.32d-h | 0.31bc | 3.166b-d | 2.70bc | 18.16a-f | 11.88fg | 0.45f-j | 0.34e-i |
| Boset | 0.31f-j | 0.29ef | 2.83d-f | 1.94e-i | 12.44h | 8.72i | 0.30k | 0.23i-k |
| RIL-302 | 0.36bc | 0.25ij | 2.00h | 1.22i | 13.35f-h | 10.88gh | 0.56d-f | 0.19k |
| Alba | 0.39a | 0.25ij | 2.836d-f | 1.21i | 17.61a-g | 16.94b-d | 0.70b-d | 0.53a-c |
| Ajora | 0.34c-e | 0.28e-g | 2.53fg | 2.00d-g | 20.00a-d | 13.22ef | 0.41g-k | 0.32f-j |
| Tsedey | 0.28k | 0.27gh | 2.54fg | 1.50e-i | 13.27f-h | 12.27fg | 0.32jk | 0.28g-k |
| Dega Tef | 0.38ab | 0.32b | 2.61e-g | 1.44g-i | 19.00a-e | 13.22ef | 0.49e-i | 0.25h-k |
| Gimbichu | 0.34c-e | 0.31b | 3.21b-d | 1.66e-i | 19.94a-d | 16.61cd | 0.76b | 0.34e-i |
| Enatite | 0.34cd | 0.30c-e | 3.21b-d | 3.11ab | 22.50a | 19.00ab | 0.42f-k | 0.42b-d |
| RIL-44 | 0.32e-i | 0.29ef | 2.47f-h | 2.05d-g | 14.16e-h | 8.88hi | 0.81b | 0.39d-g |
| RIL-94 | 0.33d-g | 0.31bc | 2.48f-h | 2.00d-h | 12.83gh | 11.66fg | 0.41g-k | 0.21jk |
| DZ-01-951 | 0.34c-e | 0.28e-g | 2.72d-f | 1.38h-i | 16.55c-h | 16.49cd | 0.35i-k | 0.41d-f |
| Dtt-2 | 0.29h-k | 0.27f-h | 3.61b | 3.27ab | 17.16b-h | 13.77ef | 0.35i-k | 0.30f-k |
| TDT-4-15 | 0.30g-k | 0.30b-e | 4.55a | 2.07de | 21.72ab | 13.55ef | 0.53e-g | 0.36e-h |
| Mean | 0.33 | 0.28 | 2.92 | 2.07 | 17.75 | 14.46 | 0.54 | 0.38 |
| LSD (5%) | 0.03 | 0.02 | 0.49 | 0.59 | 5.11 | 2.12 | 0.14 | 0.12 |

Where TKW: thousand kernel weight; GY: grain yield, SB: shoot biomass, and RB: root biomass. **Note:** mean comparison was done separately for each moisture conditions and traits. Genotype in each column designated with the same latter are not significantly different.

**Figure S1**. Temperature (^O^C) and Rainfall(mm) during the experimental period between October 2023 to January 2024 at Hawassa. Source: National Meteorology Agency (NMA) of Ethiopia.

**Figure S2.** Experimental layout of 20 tef genotypes evaluated under well-watered (WW) and water-deficit (WD) conditions using an alpha-lattice design with three replications.

**Note**: The experiment was arranged in a 5 × 4 alpha-lattice design. Each row (incomplete block), from left to right, contained five plots, while each column (incomplete block), from top to bottom, contained four plots. The three replications were arranged vertically, one above the other. The WW and WD conditions were separated by 2 m and were also arranged vertically, with the WD conditions positioned above the WW conditions. Each plot consisted of five rows spaced 0.2 m apart, with a 0.5 m distance between adjacent plots.
